# Intratumoral microbiota in the growth of CRC and lung cancer: Comprehensive insights from etiology to therapy

**DOI:** 10.1016/j.isci.2026.117254

**Published:** 2026-08-21

**Authors:** Yicheng Wang, Jixiang Li, Xuxu Du, Wutang Li, Dong Wang, Yuyuan Li

**Affiliations:** 1 The Second Hospital Affiliated to Dalian Medical University, Dalian Medical University, Dalian, China; 2 Advanced Institute for Medical Sciences, Dalian Medical University, Dalian, China

**Keywords:** colorectal cancer, lung cancer, intratumoral microbiota, gut-lung axis, cancer metastasis, microbiota-based therapy

## Abstract

The tumor microenvironment (TME) contains a diverse intratumoral microbiota (ITM) governing cancer onset, progression, and therapeutic response. This review compares ITM profiles of colorectal and lung cancer, two anatomically distinct malignancies linked via the gut-lung axis, and summarizes microbial detection strategies ranging from cultivation and sequencing to spatial and single-cell technologies. Oral pathobionts such as *Fusobacterium nucleatum*, *Streptococcus*, and *Veillonella* accumulate in both cancers, promoting tumorigenesis through chronic inflammation, immune evasion, genotoxic insults, metabolic reprogramming, and facilitating colorectal cancer lung metastasis via venous drainage and systemic dissemination. Microbial signatures in stool, saliva, blood, and tumor tissue offer non-invasive tools for detection, prognosis, and prediction of immunotherapy resistance, while antibiotics, fecal microbiota transplantation, and engineered live biotherapeutics offer strategies to remodel the TME. This synthesis positions the gut-lung axis as a systemic driver of metastasis and outlines a route toward precision microbiome-based diagnostics and therapeutics.

## Graphical abstract

**Figure:**
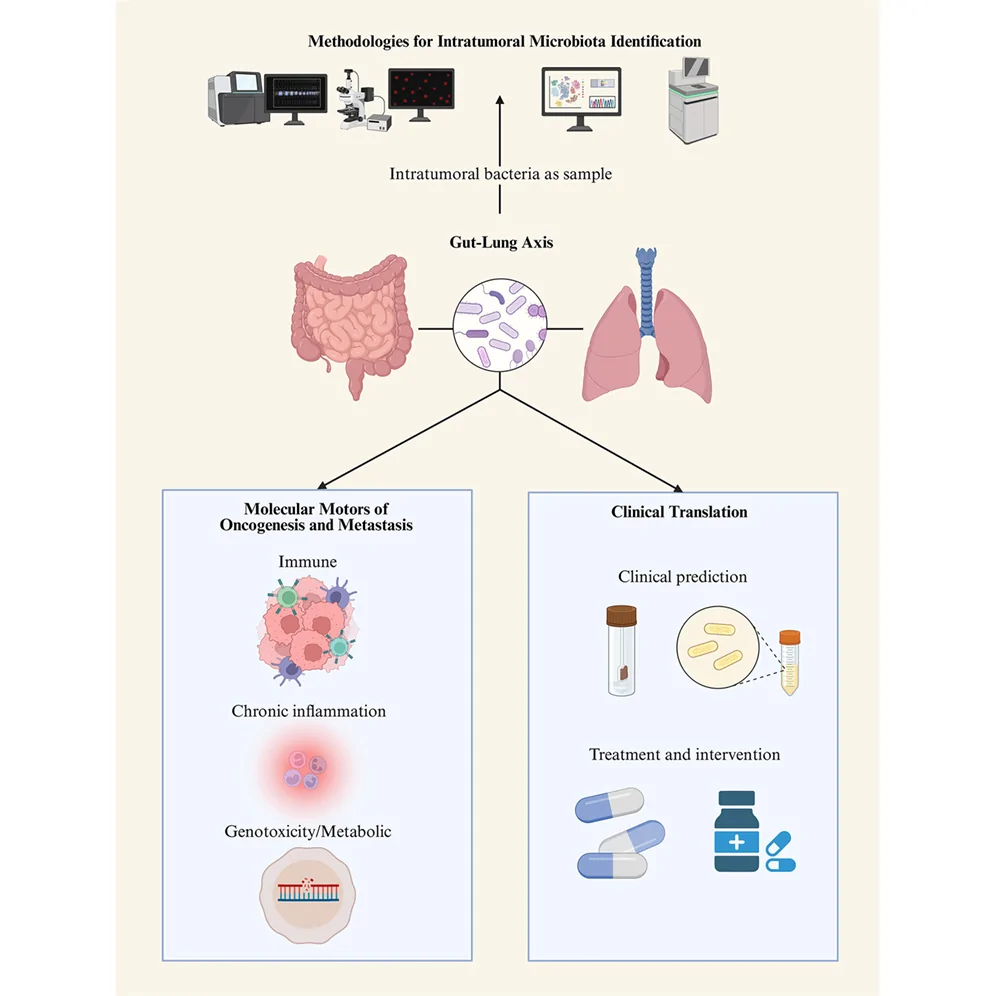

## Teaser

Microbiome; Cancer

## Introduction

Cancer is universally recognized as one of the most formidable health challenges. Its development is fueled by a spectrum of exogenous and endogenous drivers, including chemical carcinogens (e.g., tobacco nitrosamines and aflatoxin), physical agents (e.g., ionizing radiation and asbestos fibers), oncogenic viruses (e.g., HPV, HBV, and EBV), and, increasingly appreciated, bacteria that trigger chronic inflammation or deliver genotoxins.^1^^,^^2^^,^^3^ These infectious agents have been causally linked to diverse malignancies such as prostate, gallbladder, gastric, colorectal, and lung cancers.^4^^,^^5^^,^^6^^,^^7^^,^^8^ Colorectal cancer (CRC) and lung cancer consistently rank among the leading causes of morbidity and mortality worldwide.^9^^,^^10^^,^^11^ CRC accounts for 10.2% of all incident cancers and 9.2% of cancer-related deaths,^9^^,^^10^ whereas lung cancer contributes 2 million new diagnoses and 1.76 million fatalities annually.^11^ Historically, tumors were primarily viewed as aggregates of malignant epithelial cells supported by stromal elements.^12^^,^^13^ The tumor microenvironment (TME) was thus perceived as an intricate milieu composed of immune cells, vascular networks, extracellular matrix components, fibroblasts, lymphoid infiltrates, bone-marrow-derived inflammatory cells, and soluble mediators.^12^^,^^13^ This paradigm has shifted fundamentally with the recognition that the TME is a highly dynamic ecosystem that critically influences tumor fate.^14^^,^^15^^,^^16^^,^^17^ Central to this revised perspective is the intratumoral microbiota (ITM), a diverse community of bacteria, fungi, mycoplasma, viruses, and other microorganisms residing within the cancer mass.^18^^,^^19^ ITM constituents shape anti-tumor immunity, secrete bioactive metabolites, and engage in direct crosstalk with host cells. Depending on context, they can foster tumorigenesis through chronic inflammation and genotoxic stress or restrain it by reinforcing immune surveillance.^18^ By occupying privileged niches within the TME, intratumoral microbes reprogram diverse cellular compartments, modulate immune effector functions, and alter the migratory capacity of malignant epithelial cells.^19^

Once controversial, the existence of microbial communities within tumor tissue is now widely accepted across multiple malignancies, including pancreatic, breast, lung, and liver cancers. Far from being passive contaminants, these microorganisms engage in intensive molecular crosstalk with host cells, modulating proliferation, metabolism, immune surveillance, and even therapeutic resistance.^18^ CRC (long viewed through the lens of the gut microbiome) and lung cancer (traditionally anchored to the airway flora) are anatomically distant but mechanistically unified by the gut-lung axis. This bidirectional highway allows microbial cells, metabolites, and immune signals to circulate via blood and lymphatics, remotely reshaping organ-specific pathology.^20^ Consequently, intestinal dysbiosis can precondition the pulmonary microenvironment, facilitating CRC lung metastasis (CRLM), the second most common metastatic event in CRC, occurring in 20%–30% of patients.^21^^,^^22^ Importantly, the same oral-derived pathobionts, *Fusobacterium nucleatum*, *Streptococcus* spp., and *Veillonella*, are repeatedly enriched in both primary CRC and primary lung tumors, even in never-smokers.^23^^,^^24^^,^^25^^,^^26^^,^^27^ This shared microbial signature, together with the anatomical conduit described earlier, provides a natural experimental model in which identical microbes can be observed under different oxygen, immune, and metabolic pressures. Comparing their intratumoral ecology therefore reveals universal drivers that operate across organs, and identifies common therapeutic targets against either primary cancer or metastatic outgrowth.

While CRC and lung cancer are treated as clinically distinct entities, their shared microbial signatures, particularly oral-derived pathobionts like *Fusobacterium* and *Veillonella*, demand that we move beyond organ-specific paradigms. These *trans*-organ overlaps reveal a unified host-microbial ecosystem orchestrated via the gut-lung axis, where intestinal dysbiosis functions as a systemic conditioner of pulmonary metastasis. Yet, the field remains mired in descriptive profiling, lacking the spatial-functional resolution needed to decode these cross-organ dialogues. This review addresses that gap by delivering the first side-by-side, mechanism-focused atlas of ITM in CRC and lung cancer. Integrating spatial omics, fungal and viral data, and engineered live biotherapeutics, we pinpoint how identical gut-derived microbes operate in divergent microenvironments, and outline actionable strategies for precision microbiome interventions against primary tumors and organotropic metastasis.

## Methodologies for ITM identification

### Conventional identification methods: Advantages and limitations

Microbial culture, once the gold standard, still provides unequivocal evidence of viability and enables downstream functional assays such as antimicrobial susceptibility testing.^28^ Recent refinements, nutrient-rich media, anaerobic or microaerophilic atmospheres that mimic the tumor niche, and co-culture systems fortified with specific metabolites or signaling molecules, have recovered previously elusive taxa.^28^ Nevertheless, culture-dependent approaches remain intrinsically biased: the vast majority of ITM organisms exist in a “viable but non-culturable” (VBNC) state, and standard media selectively favor fast-growing or fastidious species. Consequently, true diversity, especially in low-biomass tumors such as certain lung cancer subtypes, is systematically underestimated.

To circumvent these intrinsic biases and access the full genetic repertoire of scarce or non-cultivable cells, the field rapidly embraced culture-independent molecular sequencing. Molecular sequencing has transformed the study of the intratumoral microbiome by freeing it from the constraints of culture. High-throughput, culture-independent approaches now capture taxonomic and functional information from even the scarcest, most fastidious tumor-associated microbes.^29^ Targeting conserved and hypervariable regions of the bacterial ribosome, 16S rRNA sequencing delivers rapid, cost-effective surveys of bacterial and archaeal communities. First applied to CRC tissues by Castellarin et al. in 2012,^30^ the method revealed a striking enrichment of *F. nucleatum* and launched a decade of oncogenic microbiome research. Its sensitivity allows detection of rare or unculturable taxa^31^ and has recently been exploited for pathogen diagnosis in sepsis plasma.^32^ Limitations are equally clear: fungi and viruses are invisible, resolution rarely extends beyond genus level, and no functional information is provided. Accordingly, shotgun whole-metagenome sequencing (WMS) was adopted to lift these taxonomic and functional blinds. WMS bypasses the taxonomic and functional blind spots of 16S by reading every base in the specimen, human, bacterial, fungal, and viral alike.^33^ This shotgun approach delivers species- or strain-level resolution, maps metabolic pathways and virulence factors in a single experiment, and has already uncovered broad functional dysbioses in CRC stool that serve as early-detection signatures.^34^ The price of universality is host dominance: more than 99% of reads typically originate from human DNA, forcing ultra-deep sequencing, high storage costs, and computationally intensive bioinformatics to extract the microbial signal, an obstacle that is especially acute in low-biomass tumor biopsies.^35^ Consequently, while WMS is the gold standard for profiling high-biomass samples such as feces, it is rarely applied to solid tumor tissues because overwhelming host DNA contamination (often >99%) necessitates ultra-deep sequencing or sophisticated host-depletion protocols, limiting its routine clinical use.

Because even the deepest sequencing loses spatial context, imaging platforms have become indispensable to verify true residency and to locate microbes relative to host cells and immune infiltrates. Imaging tools are indispensable for confirming that microbes truly reside within tumor tissue and for pinpointing their exact location relative to malignant and immune cells, which is a prerequisite knowledge for inferring function. Antibodies directed against microbial antigens (e.g., *F. nucleatum* adhesin Fap2) reveal the presence and topography of specific organisms in formalin-fixed sections.^36^^,^^37^ Immunohistochemistry (IHC) simultaneously displays microbial distribution and physical association with tumor cells, but its utility is constrained by the limited repertoire of high-affinity, pathogen-specific antibodies. Thus, IHC is often paired with Fluorescence *in situ* hybridization (FISH) or targeted PCR to boost sensitivity and specificity.^38^^,^^39^ FISH uses fluorescently labeled oligonucleotide probes to hybridize to microbial rRNA, enabling visualization at phylum-level resolution or down to individual species (e.g., *F. nucleatum* and *Acinetobacter* spp.).^40^^,^^41^ Because FISH targets nucleic acids, it offers superior taxonomic specificity and is critical for distinguishing genuine intratumoral residents from surface contaminants.

### Emerging technologies: Spatial and single-cell resolution

Recent breakthroughs in spatially resolved and single-cell technologies have pushed cancer-microbiome studies beyond “who is present?” to “who is where, and what are they doing?” within the tumor landscape.^42^ Because both spatial transcriptomics and HiPR-FISH physically anchor microbial signals to host histoarchitecture, they immediately reveal whether ITMs occupy immune hot spots, hypoxic cores, or invasive margins, contextual information that bulk sequencing inevitably loses. State-of-the-art platforms now co-map host and microbial nucleic acids in the same voxel: spatial host-microbiome sequencing (SHM-seq) captures genomes plus transcriptomes, whereas spatial *meta*-transcriptomics (SmT) snapshots active metabolism and instantaneous host responses.^43^^,^^44^ High-throughput HiPR-FISH goes further, creating micrometre-scale atlases of >200 taxa and quantifying physical contacts between microbes and T cells, macrophages, or malignant cells, thereby exposing the social network of the intratumoral microbiome.^45^^,^^46^

While these imaging approaches paint the neighborhood, single-cell RNA platforms resolve the individual residents. Single-cell sequencing (SCS) mines microbial reads stably associated with dissociated tumor or immune cells, yielding indirect but high-resolution portraits of which phenotypes malignant cells, M2 macrophages or exhausted T cells, preferentially harbor or respond to ITM components.^47^ INVADE-seq streamlines this marriage by simultaneously capturing eukaryotic poly-A RNA and bacterial 16S rRNA in one library, allowing downstream pathways of immune subversion or metabolic reprogramming to be mapped specifically within microbe-positive subpopulations.^19^^,^^48^

Yet every platform described above has so far been deployed within a single cancer type; a systematic, side-by-side spatial, or single-cell map comparing CRC and lung cancer, two anatomically distant but connected via the gut-lung-axis malignancies, remains conspicuously absent. Closing this comparative gap is essential if we are to graduate from descriptive atlases to function-driven, precision microbiome therapeutics.

### Bioinformatic challenges: Contamination control and standardization

**Figure 1:**
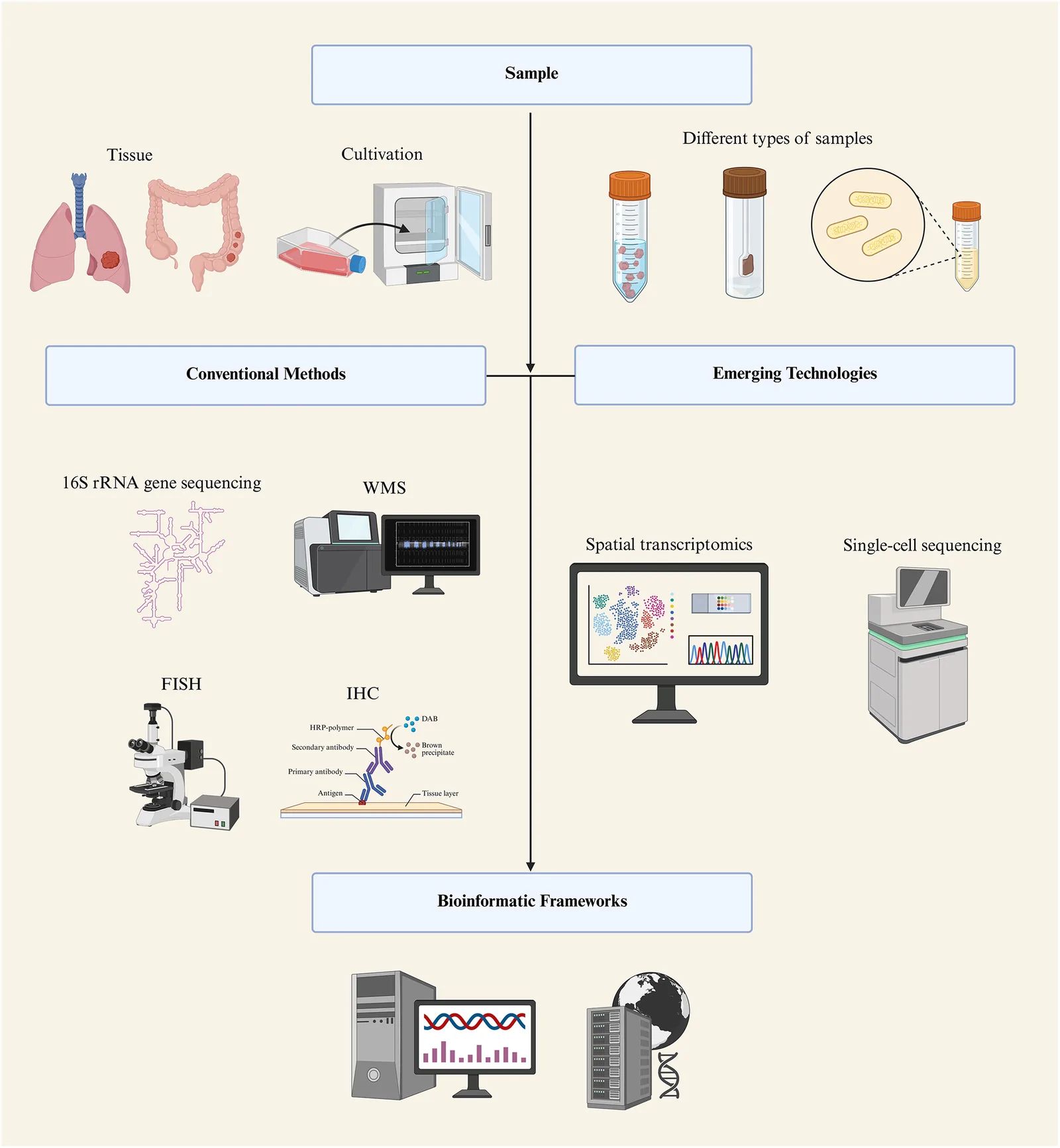
Multimodal detection of intratumoral microbiota Overview of ITM identification methods: specimens (tissue, blood, and body fluids) are interrogated by conventional tools (16S rRNA, WMS, FISH, and IHC) and emerging spatial or single-cell platforms, with all data streams converging into a unified bioinformatic pipeline for contamination-aware, high-resolution analysis.

Rigorous interpretation of the intratumoral microbiome remains shackled by the extreme scarcity of microbial DNA within tumor specimens, a circumstance that magnifies the influence of every contaminant molecule originating from reagents, laboratory surfaces, or operator skin. These exogenous sequences, collectively dubbed the “kitome,” can readily masquerade as authentic tumor microbes, giving rise to spurious community profiles that fracture reproducibility across studies.^28^^,^^49^ Complicating matters further is the absence of methodological uniformity: variations in tissue handling protocols, DNA-extraction chemistries, primer choice for marker-gene amplification, sequencing depth, and downstream analytic pipelines each introduce their own layer of technical noise, so that no two datasets are directly comparable.^29^ To escape this cycle of conflicting signals, future investigations must embed an armada of negative controls, reagent blanks, collection devices, and adjacent normal tissue, alongside every batch of tumor biopsies, and must adopt community-accepted minimum reporting standards that transparently document each analytical decision. Only when contamination-aware bioinformatics dynamically strips away kit-derived reads while safeguarding bona-fide, low-abundance tumor microbes will the intratumoral microbiome emerge as a credible and actionable therapeutic target. To fully appreciate the capabilities and limitations of ITM research, we provide a comprehensive overview of current multimodal detection strategies, ranging from traditional cultivation methods to cutting-edge high-resolution spatial sequencing technologies, and illustrate their respective roles in mapping the tumor microbiome (as shown in Figure 1). Given the inherently low biomass of ITM and the necessity for cross-platform validation, we critically summarize the advantages, inherent limitations, and suitable application contexts of each method in Table 1.

**Table 1:** The advantages, disadvantages, and applicable scenarios of different detection methods

	Method	Advantages	Disadvantages	Application scenarios	Reference
Conventional methods	cultivation techniques	enables isolation and purification of microorganisms	difficult to culture most intratumoral microorganisms	microbial isolation and purification	Lagier et al.^50^; Kapinusova et al.^51^; Yan et al.^52^; Wan et al.^53^
		suitable for functional validation and drug sensitivity testing	low microbial abundance, challenging to detect	functional validation	
		allows study of microbial biological characteristics	results biased by cultivation conditions	drug sensitivity testing	
	16S rRNA gene sequencing	detects rare or unculturable microorganisms	limited to bacteria and archaea, cannot detect fungi and viruses	studying microbial diversity and community structure	Lane et al.^54^; Church et al.^55^; Janda and Abbott^56^; Osman et al.^57^
		efficiently reveals microbial community structure	relies on databases, may miss new microorganisms	detecting bacteria and archaea	
		suitable for large-scale studies	no functional information	large-scale microbial surveys	
	whole metagenome sequencing (WMS)	provides comprehensive taxonomic and functional information	high cost	indepth study of microbial genomic structure and function	Tringe and Rubin^58^; Schloss and Handelsman^59^; Liu et al.^60^
		detects non-bacterial microorganisms (e.g., fungi and viruses)	complex data analysis, requires substantial computational resources	detecting non-bacterial microorganisms	
		reveals metabolic pathways and host interactions	host DNA interference reduces sensitivity; complex bioinformatics; not optimal for low-biomass tumor biopsies unless coupled with enrichment or host-depletion strategies	studying microbial-host interactions	
Imaging-based identification	immunohistochemistry (IHC)	visually displays microbial distribution in tissues	sensitivity depends on antibody specificity and affinity	studying local distribution and qualitative analysis of microorganisms	Tan et al.^61^; Harms et al.^62^; Magaki et al.^63^
		reveals physical positional relationships between microorganisms and tumor cells	developing efficient antibodies is challenging	observing interactions between microorganisms and tumor cells	
		relatively simple to perform	limited detection of low-abundance microorganisms	–	
	fluorescence *in situ* hybridization (FISH)	high specificity, detects changes at the nucleic acid level	requires high level of technical expertise	studying spatial distribution and co-existence patterns of microorganisms	Prudent and Raoult^40^; Frickmann et al.^64^; Kempf et al.^65^; Kostic et al.^66^
		simultaneously detects multiple microorganisms	complex probe design, difficult for multi-species detection	detecting multiple microorganisms (e.g., bacteria, viruses, fungi)	
		directly localizes microorganisms in tissues	challenging to detect low abundance microorganisms	–	–
Emerging high-resolution technologies	spatial transcriptomics	reveal spatial, cellular, and molecular host-microbe interactions	the high expense of platforms	niche identification	Galeano Niño et al.^19^; Emens et al.^67^; Galeano Niño et al.^68^
				local functional impact	
	single-cell sequencing	providing high-resolution insights into cellular interactions and microbial presence within tumors	the high expense of platforms	interaction phenotypes	Emens et al.^67^; Lu et al.^47^
		sequencing aids in elucidating the roles of specific subsets of immune and tumor cells influenced by microbiota	–	cellular microbe reservoir	

While sequencing has eclipsed culture-based methods, it has imported a reproducibility crisis, especially for low-biomass tumor samples where the “kitome,” contaminant DNA from reagents, masquerades as authentic signal. We stress that presence alone is no longer scientifically sufficient; future work must anchor microbial detection to rigorous negative controls and spatial validation. Only by separating genuine intratumoral residents from laboratory noise can we forge clinically actionable biomarkers.

## Comparative ecology of ITM in CRC and lung cancer: Bacteria, fungi, and viruses

Comparative ecology of the ITM in CRC and lung cancer reveals that microbial colonization is anything but random: tissue oxygen, pH, nutrient gradients, and anatomical distance to major reservoirs jointly select for distinct yet functionally convergent communities.^69^^,^^70^^,^^71^ In CRC, the gut lumen acts as the primary inoculum, whereas the aerodigestive tract feeds the lung tumor niche; nevertheless, both malignancies share overlapping pathobiont signatures that extend beyond bacteria to fungi and viruses.

### Core bacterial signature of CRC

Across multiple cohorts, CRC mucosa exhibits a consistent loss of α-diversity and a parallel bloom of pro-oncogenic taxa; global comparisons show reduced within-sample richness but increased between-sample dissimilarity relative to matched normal mucosa.^72^ This community collapse is dominated by a phylogenetic swap: expansion of *Fusobacteriota* and *Proteobacteria* at the expense of *Bacteroidetes*, *Firmicutes*, *Verrucomicrobia*, and *Actinobacteria*.^17^^,^^73^ Within this shifted landscape, *F. nucleatum* acts as the flagship pathobiont. Via its surface adhesin FadA, it binds epithelial E-cadherin, unleashes β-catenin signaling and accelerates proliferation; concurrently, its interaction with the DEAH-box helicase DHX15 specifically hastens progression in KRAS p.G12D-mutant tumors.^73^ Enrichment is not diffuse but anatomically hierarchical: *F. nucleatum* density is highest in the tumor core, lower at the margin, and almost undetectable in distant mucosa, and this gradient correlates consistently with advanced stage, lymph-node metastasis and shorter survival.^30^^,^^74^ Alongside *F. nucleatum*, a core dysbiotic consortium emerges: *Parvimonas micra*, enterotoxigenic *Bacteroides fragilis* (ETBF), *Peptostreptococcus* spp., and *Prevotella intermedia*.^75^ They complement this assault by secreting BFT, a metalloprotease that cleaves E-cadherin and triggers sustained Wnt/β-catenin and NF-κB signaling, fueling uncontrolled growth and chronic inflammation.^76^ pks^+^ *E. coli* delivers the genotoxin colibactin, which alkylates DNA, produces double-strand breaks and activates ATM-mediated pro-survival signaling, locking cells into a mutator phenotype.^76^ *Streptococcus gallolyticus* deploys the Pil1 pilus to ligate collagen/integrin α2β1, triggering STAT3 and COX-2 cascades that support tumorigenesis.^77^ Counter-balancing this pathobiont bloom, protective commensals such as *Clostridium butyricum* and *Streptococcus thermophilus*, which antagonize Wnt/β-catenin signaling and reinforce barrier integrity, are depleted, tilting the microenvironment toward a tumor-permissive state.^78^

### Unique characteristics of ITM in lung cancer

Unlike the gut-rich milieu of CRC, lung parenchyma is physiologically low-biomass but still harbors a baseline airway microbiota that includes oral-commensal taxa such as *Streptococcus*, *Veillonella*, and occasionally *Fusobacterium*, whose numbers can surge within the TME independently of external seeding.^44^^,^^71^^,^^79^ This resident bloom is fueled by cancer-specific stresses: regional hypoxia, acidic pH, nutrient patches, and impaired mucociliary clearance, can trigger local expansion of these resident microbes, independent of any external seeding. Superimposed on these local dynamics are two continuous inocula, micro-aspirated oropharyngeal secretions and hematogenously delivered gut-derived bacteria hitchhiking on circulating tumor cells or exosomes, so that malignant tissue frequently carries a higher bacterial load than paired normal parenchyma.^71^ Disentangling airway-resident expansion from distal translocation is now an experimental priority, and spatial or isotope-tracing studies are being designed to meet it.^44^

Across histological subtypes, the same ecological drivers generate a reproducible signature: adenocarcinomas and squamous cell carcinomas alike exhibit an outgrowth of *Proteobacteria* and *Firmicutes*, with *Pseudomonas*, *Acinetobacter*, *Veillonella*, and *Streptococcus* consistently enriched relative to control airways.^80^^,^^81^^,^^82^ Parallel profiling of saliva and sputum reveals elevated *Granulicatella*, *Abiotrophia*, and oral *Streptococcus*, underscoring the oral-bronchial axis as an additional, but not exclusive, source.^83^ *Pseudomonas* predominates in many tissue series, yet whether it actively drives oncogenesis or simply exploits a tumor-permissive niche remains under active investigation.^84^^,^^85^

Histology and smoking history further sculpt the ecosystem. *Veillonella* and *Streptococcus* are frequently linked to the inflammatory background of squamous-cell carcinoma, whereas specific *Pseudomonas* lineages suggest chronic airway colonization.^80^^,^^86^^,^^87^ Advanced-stage tumors (IIIB/IV) enrich for *Thermus*, and *Legionella* is over-represented in metastatic lesions, while non-malignant parenchyma retains higher overall diversity.^79^ Importantly, the ITM profile diverges from concurrent bronchoalveolar lavage fluid, implying selective retention or adaptation within the TME rather than passive reflection of airway contents, an observation consistent with both resident-microbe expansion and exogenous seeding as cooperative, non-mutually exclusive mechanisms shaping the lung-cancer microbiome.

### Non-bacterial ITM: The mycobiota and virobiota

Bacteria alone tell only half of the story. Fungi and viruses, collectively the mycobiota and virobiota, form a vast ecological layer that shape oncogenesis and the therapeutic response. Fungal communities inside colorectal tumors differ sharply from those of adjacent mucosa. Early adenomas are dominated by *Ascomycota* (43%), whereas invasive carcinomas show a surge of *Basidiomycota* and a net loss of fungal α-diversity.^69^^,^^88^ *Malassezia globosa*, a skin commensal repeatedly recovered from CRC tissue, can activate MAPK and PI3K-Akt cascades, accelerating epithelial proliferation.^89^^,^^90^^,^^91^^,^^92^ *Candida albicans*, when over-represented, amplifies neutrophilic inflammation and fosters a tumor-permissive microenvironment. Because fungal cells are larger and morphologically distinct, their accurate detection demands dedicated DNA-extraction protocols, long-read sequencing, and taxonomically curated databases. The viral fraction includes both oncogenic eukaryotic viruses and an enormous pool of bacteriophages. Latent Epstein-Barr virus or polyomavirus DNA is occasionally detected in CRC and lung tumors, where they may act as cofactors that potentiate carcinogenic signaling. The dominant virobiota component, however, is bacteriophages. By lysing or lysogenizing their bacterial hosts, phages govern ITM population dynamics and can horizontally transfer virulence or antibiotic-resistance genes across bacterial lineages within the tumor milieu. Thus, the virobiota not only mirrors but actively sculpts the pathogenic potential of the bacterial ITM.

### Niche adaptation and functional convergence of shared taxa

Despite their anatomical separation, CRC and lung tumors converge on a strikingly similar ITM dominated by *Fusobacterium*, *Porphyromonas*, *Streptococcus*, *Veillonella*, genera that overwhelmingly originate in the oral cavity or gut.^83^^,^^93^ This *trans*-organ recurrence reflects powerful evolutionary convergence: after surviving hematogenous or lymphatic dissemination, these microbes must evade systemic immunity, withstand oxidative stress, hypoxia, and nutrient scarcity, and ultimately exploit the tumor as a new ecological niche. *F. nucleatum* exemplifies the resulting niche adaptation: beyond binding to E-cadherin and TLR4, it up-regulates oxidative-stress enzymes, scavenges tumor-derived nutrients and cloaks itself in host proteins to escape neutrophil killing, strategies that license colonization of both colonic and pulmonary tissue. Under the same selective pressures, *Streptococcus* spp. express heme-acquisition systems while *Veillonella* ferments lactate secreted by cancer cells, ensuring persistence far from their commensal reservoirs.

Functional convergence follows inevitably from these shared survival tactics. fusobacterial Fap2, streptococcal hemolysins, and veillonellal lipopolysaccharides all activate NF-κB/STAT3 signaling, fueling chronic inflammation, generating genotoxic reactive species and blunting cytotoxic T cell infiltration.^94^^,^^95^^,^^96^ The net effect is a similarly tumor-permissive microenvironment in colon and lung, underscoring the systemic reach of the gut-lung axis and explaining why intestinal dysbiosis can remotely sculpt pulmonary metastatic niches. Table 2 summarizes this head-to-head taxonomic and functional convergence across the two malignancies.

**Table 2:** Changes in the types and numbers of microorganisms in colorectal and lung cancers

Cancer types	Associated microbiota	Quantitative dynamics	Reference
Colorectal cancer	*Enterotoxigenic Bacteroides fragilis*, *Fusobacterium*, *Lactococcus*, *Bacteroides*, *Prevotella*, *Streptococcus*, *Fusobacterium nucleatum*, *Bifidobacterium*, *Romboutsia*, *Haemophilus*, *Veillonella*, *Ascomycota*, *Malasseziomycetes*, *Escherichia coli*, *Enteropathogenic Escherichia coli*, *Hungatella hatheway*, *Akkermansia*, *Candida*	increase	Kostic et al.^66^; Coker et al.^91^; Goodwin et al.^97^; Dejea et al.^98^; Chung et al.^99^; Zhou et al.^100^; Gao et al.^101^; Chen et al.^102^; Yamamoto et al.^103^; Guo et al.^104^; Park et al.^105^; Hong et al.^106^; Xia et al.^107^; Koi et al.^108^; Tang et al.^109^; Borowsky et al.^110^; Gur et al.^111^^,^^112^; Wu et al.^113^; Zhang et al.^114^; Kneis et al.^115^; Bertocchi et al.^116^; Maddocks et al.^117^; Triner et al.^118^; Dohlman et al.^119^
Colorectal cancer	*Pseudomonas*, *Escherichia-Shigella*, *Saccharomycetes*, *Pneumocystidomycetes*	decrease	Coker et al.^91^; Gao et al.^101^
Lung cancer	*Modestobacter*, *Blastomyces*, *Agaricomycetes*, *Aspergillus*, *Acidovorax*, *Klebsiella*, *Anaerococcus*, *Acinetobacter*, *Brevundimonas*, *Propionibacterium*, *Cyanobacteria*, *Veillonella*, *Megasphaera*, *Coriobacteriaceae*, *Pasteurella*, *Nontypeable Haemophilus influenzae*	increase	Gomes et al.^86^; Dohlman et al.^119^; Mao et al.^120^; Narunsky-Haziza et al.^121^; Greathouse et al.^122^; Apopa et al.^123^; Lee et al.^124^; Zheng et al.^125^; Jungnickel et al.^126^
Lung cancer	*Propionibacterium*, *Enterobacteriaceae*	decrease	Mao et al.^120^

## Molecular motors of oncogenesis and metastasis: Metabolism, immunity, and genotoxicity

### Anatomical conduits for systemic dissemination: Location, venous drainage, and the gut-lung axis

ITM is not a silent passenger; it reprograms host signaling pathways at every level, from systemic metabolism to single-cell DNA repair, thereby accelerating both primary growth and distant seeding. To understand how ITM fuels metastasis, we must first trace the anatomical routes by which microbes exit the primary tumor and reach distant organs. For microbes to sculpt metastatic niches, they must first escape the primary lesion and reach the circulation. Intracellular *F. nucleatum* can co-travel with circulating tumor cells or be packaged into exosomes that enter the bloodstream.^127^^,^^128^^,^^129^ Once in the vasculature, their downstream fate is dictated by vascular anatomy.

In CRC, venous drainage patterns critically determine metastatic tropism. Proximal tumors empty into the superior mesenteric vein; distal colon and upper rectum join the inferior mesenteric vein, both routes converge on the portal vein and therefore on the liver. In contrast, the middle and inferior rectal veins bypass the portal system, emptying directly into the inferior vena cava and thus delivering rectal-derived bacteria, metabolites and CTC-microbe complexes to the lung without hepatic filtration.^130^^,^^131^^,^^132^ This non-portal shortcut provides an anatomical explanation for why rectal primaries contribute disproportionately to the 5%–15% of CRC patients who ultimately develop CRLM.

**Figure 2:**
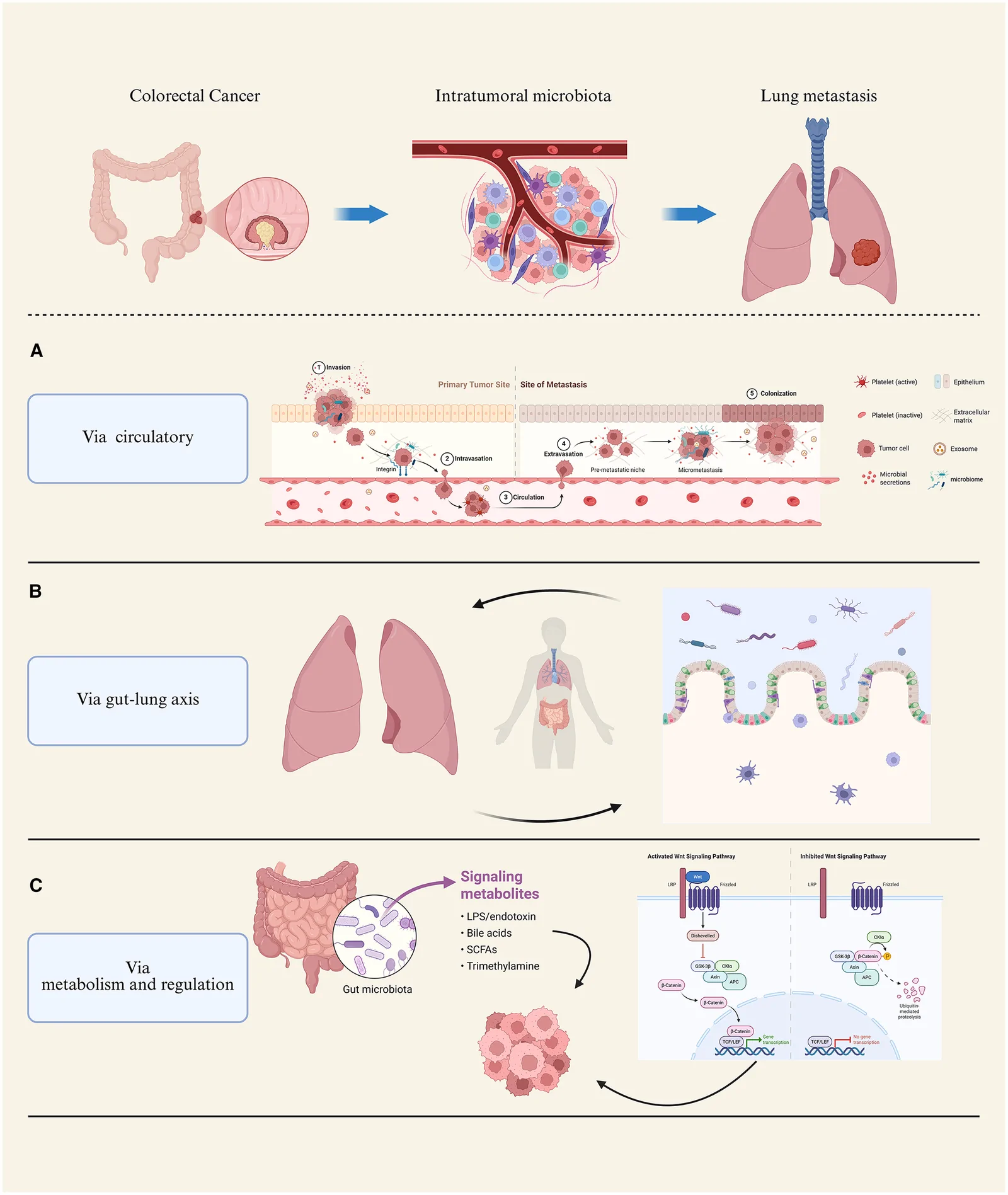
Intratumoral microbiota promote CRC lung metastasis via systemic dissemination, gut-lung axis, and metabolite-mediated signaling (A) Hematogenous spread: tumor-associated microbes enter circulation and seed the lungs. (B) Gut-lung axis: microbial signals (e.g., LPS, bile acids, and SCFAs) translocate from gut to lung, modulating the metastatic niche. (C) Metabolic reprogramming: bacterial metabolites such as trimethylamine (TMA) further regulate metastatic progression.

Beyond anatomy, the gut-lung axis also functions as an active molecular highway. Dysbiotic gut pathobionts release LPS, flagellin, and oncogenic bile acids that enter the systemic circulation, while gut-primed T cells and dendritic cells traffic to the pulmonary interstitium. Together these signals establish a pro-metastatic microenvironment characterized by chronic IL-6/STAT3 activation, PD-L1 up-regulation and suppressed NK-cell cytotoxicity.^127^^,^^129^^,^^133^ *In situ* hybridization confirms *F. nucleatum* co-localizing with malignant cells within lung lesions, underscoring that the same organism driving primary CRC can translocate from the rectal mucosa to the pulmonary metastatic niche.^129^^,^^134^ Crucially, the converging lines of evidence formally elevate the gut-lung axis from a hypothetical construct to a functional inter-organ communication route. The stepwise cascade, whereby intestinal dysbiosis remotely reprograms the pulmonary immune landscape and licenses metastatic seeding, is dissected in mechanistic detail in the conceptual model (Figure 2).

### ITM-driven reprogramming of the TME and immune escape

ITM functions as a systemic architect of TME, commandeering host immunity to erect a sanctuary for malignant progression.^16^^,^^135^ Building on this overarching immune-editing capacity, we now dissect the sequential tactics pathogens employ: first dismantling anti-tumor defense, then sustaining smouldering inflammation.

Pathobionts actively convert the TME into an immunologically privileged site. *F. nucleatum*, a flagship offender, uses its surface adhesin Fap2 to latch onto TIGIT on NK and T cells, instantly blocking cytotoxic responses.^98^^,^^111^^,^^136^ In CRC, adherent-invasive *E. coli* and *F. nucleatum* operate synergistically: the latter engages both E-cadherin and TLR4, short-circuiting cytotoxic T cell circuits while accelerating tumor growth.^137^^,^^138^ These signals expand regulatory T cells (Tregs) and myeloid-derived suppressor cells (MDSCs), further entrenching immune tolerance.^133^^,^^139^ Conversely, probiotic *Lactobacillus-Bifidobacterium* consortia restore butyrate-rich SCFA landscapes, epigenetically restraining angiogenesis while rearming NK-cell cytotoxicity, thereby blunting metastatic seeding.^139^^,^^140^

**Figure 3:**
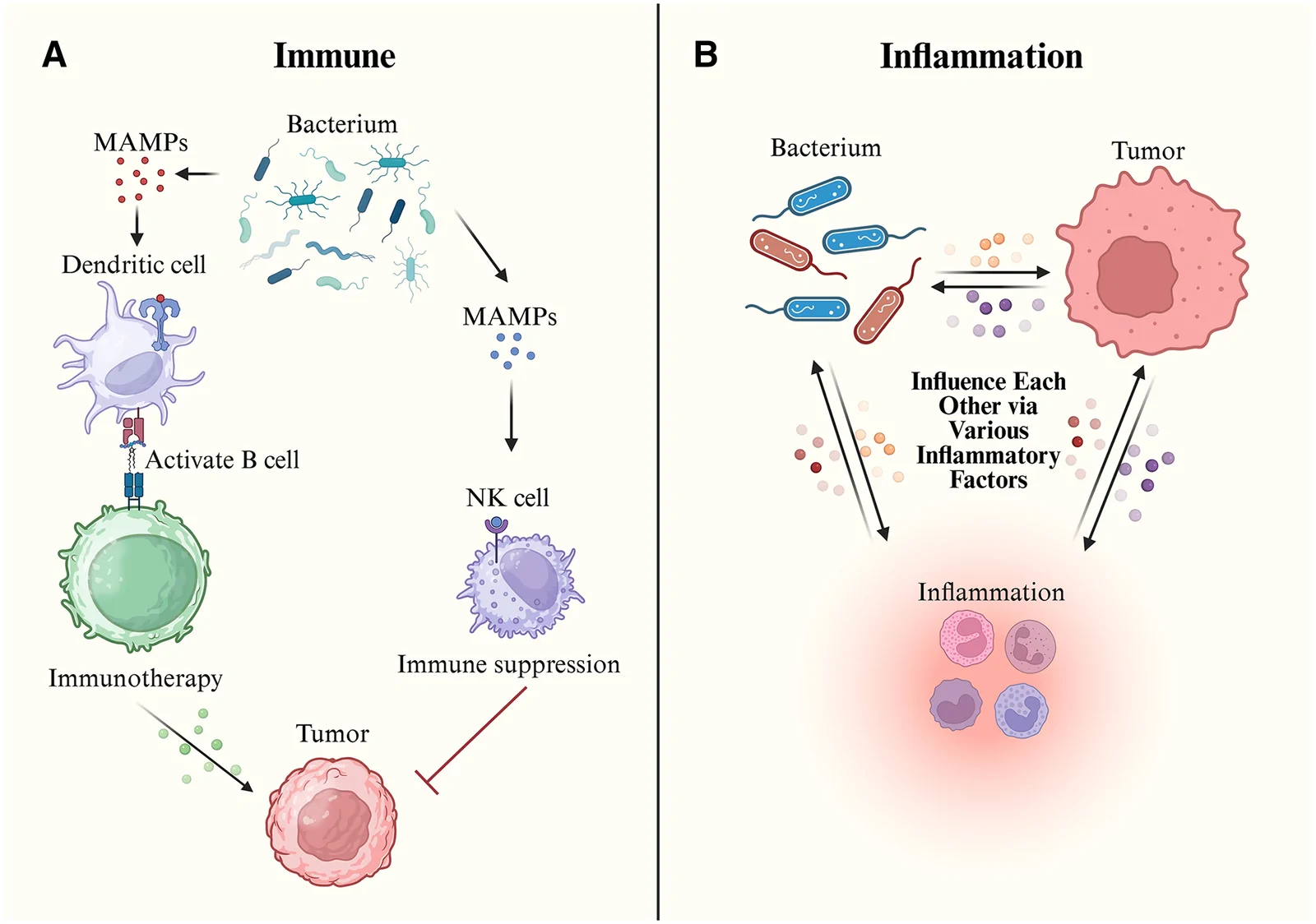
ITM-driven immune reprogramming and chronic inflammation in CRC and lung cancer (A) Immune subversion: bacterial MAMPs engage pattern recognition receptors on dendritic cells (DCs), B cells, and NK cells, triggering either tolerogenic DC maturation (amplifying regulatory B cells) or direct NK cell paralysis, thereby dismantling anti-tumor immunity. (B) Inflammatory feedforward loop: pathobionts secrete pro-inflammatory effectors (e.g., LPS and BFT) that remodel the TME, which in turn selects for microbial colonization, creating a self-perpetuating oncogenic circuit.

Immune paralysis is only the opening gambit; persistent, low-grade inflammation is the ITM’s most potent carcinogenic currency. Pattern-recognition-receptor (PRR) signaling translates microbial presence into a mutagenic inflammatory program: genotoxins such as colibactin and cytolethal distending toxin introduce DNA double-strand breaks, while LPS-TLR4-driven NF-κB activation locks epithelial and immune compartments into a cytokine-rich state that favors survival, proliferation and epithelial-to-mesenchymal transition.^137^^,^^141^ *F. nucleatum* crystallizes this paradigm by stabilizing β-catenin via E-cadherin binding, creating an inflammatory feedforward loop that sustains uncontrolled epithelial expansion.^137^ In the lung, *Veillonella* and *Prevotella* secrete matrix remodeling, pro-inflammatory metabolites that heighten tumor-cell motility.^80^^,^^141^^,^^142^ Collectively, ETBF and *F. nucleatum* maintain the smouldering inflammation that underpins both primary tumorigenesis and distant metastasis. Figure 3 summarizes this conserved triad, ITM first ignites chronic PRR-driven inflammation, then actively disable cytotoxic T cells and NK cells, tipping the immune balance irrevocably toward tumor progression.

### Metabolic sabotage and genotoxic insults

In addition to immune hijacking, the ITM wields a second, equally potent arsenal: direct DNA damage and metabolic reprogramming that remodel the TME from within. Living mutagens such as ETBF secrete BFT, a metalloprotease that cleaves E-cadherin and triggers DNA double-strand breaks in colonic epithelia, while pks^+^ *E. coli* and *Campylobacter/Helicobacter* deliver colibactin or cytolethal distending toxin that nick host DNA and activate ATM-mediated pro-survival signaling, accelerating mutation acquisition.^76^ Pathobiont-generated ROS flood the milieu, oxidizing guanine and fueling G→T transversions characteristic of both CRC and lung cancer, thereby imposing a microbiome-driven “mutator phenotype.”^16^^,^^76^

**Figure 4:**
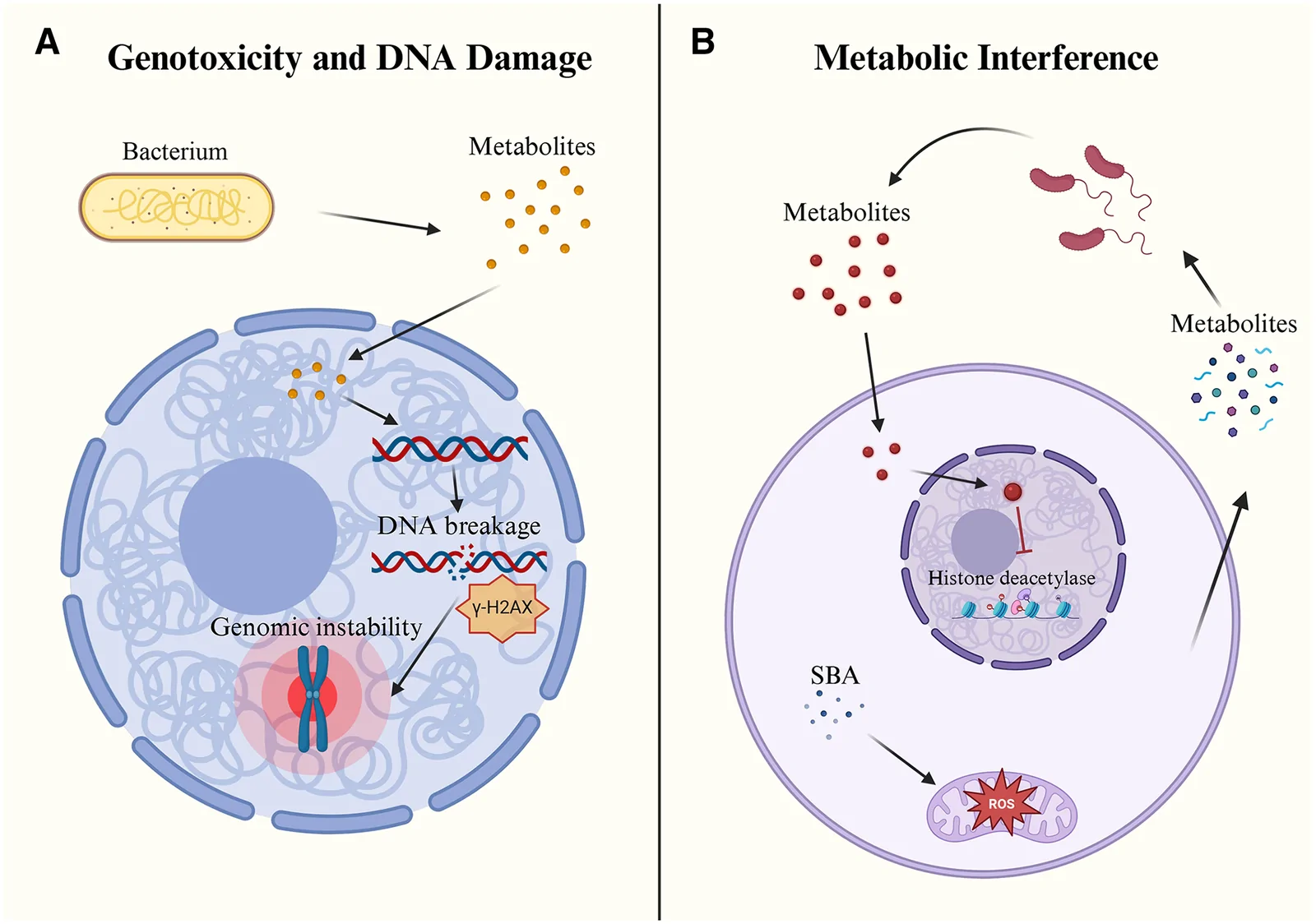
ITM-driven genotoxicity and metabolic interference in CRC and lung cancer (A) Direct genotoxic assault: microbial genotoxins (e.g., colibactin) penetrate the nuclear envelope, inducing DNA double-strand breaks and chromosomal instability—imposing a microbiome-driven mutator phenotype. (B) Bidirectional metabolic hijacking: bacterial metabolites inhibit host histone deacetylases and mitochondrial function, while simultaneously exploiting host-derived nutrients, rewiring cellular metabolism toward tumor fitness.

Parallel to this genotoxic assault, microbial metabolites act as diffusible oncogenic hormones. Butyrate, the canonical HDAC inhibitor that maintains epithelial integrity in the healthy colon, switches to a prolipogenic, proangiogenic substrate within established tumors or when ectopically elevated in lung tissue; conversely, probiotic *Lactobacillus-Bifidobacterium* consortia restore physiological butyrate levels, epigenetically silence VEGF and rearm NK-cell cytotoxicity, thereby restraining metastatic seeding.^76^ Microbial hydroxysteroid dehydrogenases convert primary bile acids into genotoxic secondary species such as deoxycholic acid, which ligate FXR/TGR5 to unleash NF-κB-driven inflammation and ROS production that propel colorectal carcinogenesis.^76^ Likewise, microbial choline and L-carnitine catabolism yields trimethylamine, oxidized by hepatic FMO3 to trimethylamine-N-oxide (TMAO); elevated TMAO rewires cellular bioenergetics, heightens metastatic fitness and correlates with advanced-stage dissemination.^143^^,^^144^ Collectively, these genotoxins and oncometabolites convert distant organs into pre-metastatic niches long before physical metastasis occurs. Figure 4 integrates these multifaceted pathways, from DNA double-strand breaks to metabolic rewiring that locks cancer cells into oncogenic addiction, into a unified mechanistic landscape.

## From bench to bedside: Translating the ITM into diagnostics, prognostics, and therapeutics

### ITM as a multi-compartment biomarker engine

Next-generation sequencing has turned the cancer-associated microbiome into a real-time, cross-compartment reservoir of diagnostic signals.^145^ For CRC, a fecal classifier that combines *F. nucleatum*, *Parvimonas micra*, and ETBF while depleting butyrate producers such as *C. butyricum* yields >90% sensitivity for stage I-II lesions and outperforms fecal immunochemical testing.^78^^,^^146^ In lung cancer, a saliva signature dominated by *Veillonella* and *Capnocytophaga* distinguishes patients from controls with an AUC of ∼0.85,^147^ and bronchoalveolar lavage fluid enrichment in *Veillonella*, *Megasphaera* and TM7 scales with tumor diameter and nodal burden.^124^ Both stool and saliva can be collected at home, providing scalable, noninvasive tools for population-level screening of high-risk individuals.

**Figure 5:**
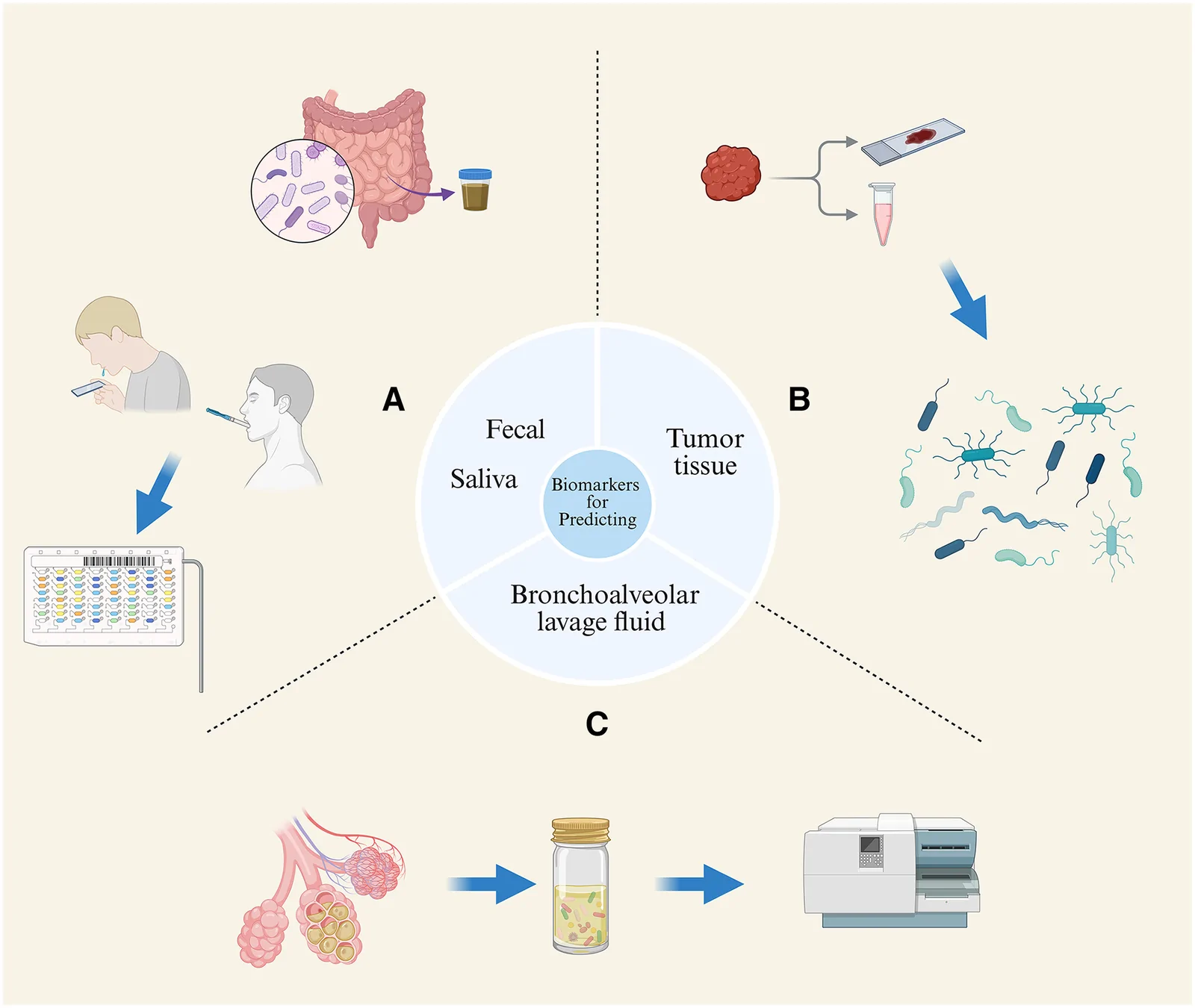
Multi-specimen microbiota profiling for predicting CRC, lung cancer, and CRC lung metastasis (A) Non-invasive samples: fecal and salivary microbiota. (B) Tumor tissue: intratumoral microbiota. (C) BALF: bronchoalveolar lavage fluid microbiota.

Tissue-level analyses show that a high intratumoral *F. nucleatum* load independently predicts shorter overall survival (HR ≈ 1.8), earlier recurrence and poorer chemotherapy response in CRC, while low α-diversity coupled with *Streptococcus*/*Pseudomonas* dominance in resected lung tissue signals accelerated post-operative relapse.^78^^,^^148^ Complementing these tissue signatures, plasma cmDNA enriched for *Fusobacterium*-specific 16S sequences can be detected months before radiological metastasis becomes apparent, and concomitant depletion of butyrate-producer cmDNA identifies minimal residual disease with >92% negative predictive value.^114^^,^^149^ Blending cmDNA quantification with conventional CEA or ctDNA metrics yields composite recurrence-risk scores that are now entering prospective validation trials, underscoring that robust, multi-specimen microbial profiles, from feces and saliva to tumor tissue and BALF, are poised to deliver actionable, timeline-sensitive forecasts of disease risk and metastatic spread (Figure 5).

### Therapeutic strategies: Rewiring the ITM-immunity axis

ITM is not merely a prognostic footnote; it is a therapeutically pliable driver of cancer progression and drug response. Rather than relying on wholesale community transfers, current strategies seek to rewire the TME in three sequential steps: first, silence pathobionts; next, reinstall immune-supportive taxa; finally, deploy programmable microbes that deliver oncolytic or immunostimulatory payloads on demand.

Checkpoint modulation exemplifies step one. Across CRC and lung cancer cohorts, an ITM dominated by *F. nucleatum* or specific *Bacteroides* spp. foretells anti-PD-1 resistance: Fap2-mediated TIGIT engagement paralyzes dendritic and T cells while β-catenin signaling accelerates tumor growth, jointly ushering in terminal exhaustion.^74^ Conversely, enrichment with *Bifidobacterium* spp. or *Akkermansia muciniphila* predicts durable benefit; after a simple oral gavage these commensals traffic to the tumor bed, expand CD8^+^ T cell infiltrates and elevate IFN-γ signatures, converting “cold” lesions into inflamed targets.^150^^,^^151^ Perioperative randomized trials show that *Lactobacillus rhamnosus* GG reduces postsurgical infections and enhances 5-FU tolerability in CRC, whereas *Akkermansia* supplementation restores Th1 immunity and prolongs progression-free survival in metastatic disease.^152^^,^^153^^,^^154^ Translational protocols now couple brief, pathobiont-directed antibiotics with fecal microbiota transplantation from ICI-complete responders to “preprogram” the TME before the first checkpoint infusion; yet this synergy is fragile, indiscriminate antibiotics, high-fat diets or untreated periodontal disease can rapidly erode beneficial taxa and re-establish ICI refractoriness.^152^^,^^155^

With pathobionts suppressed, step two reseeds the ecosystem. Short-course metronidazole or azithromycin selectively collapses *F. nucleatum* biofilms in primary CRC and matched lung metastases, synergizes with oxaliplatin or gefitinib without global dysbiosis, and is safely localized to the invasive front by FISH.^19^^,^^129^ Nevertheless, prolonged broad-spectrum coverage is deliberately avoided because it annihilates beneficial butyrate producers and has been linked to accelerated checkpoint-blockade resistance and even disease progression in some preclinical models.^156^^,^^157^ Once the pathogen load is reduced, rigorously screened donor fecal microbiota transplantation reconstitutes butyrate-producing commensals, lowers systemic IL-6 and trimethylamine N-oxide levels, and delays colorectal liver-metastasis progression in pilot trials; a single colonoscopic FMT followed by oral capsules decrease *F. nucleatum* abundance in both gut lumen and distant lung lesions and correlates with prolonged progression-free survival.^158^^,^^159^^,^^160^ Thus, antibiotics and FMT function not as competing tactics but as sequential instruments, first ‘silence the villains’ (pathobionts), then ‘reinstall the guardians’ (commensals).

**Figure 6:**
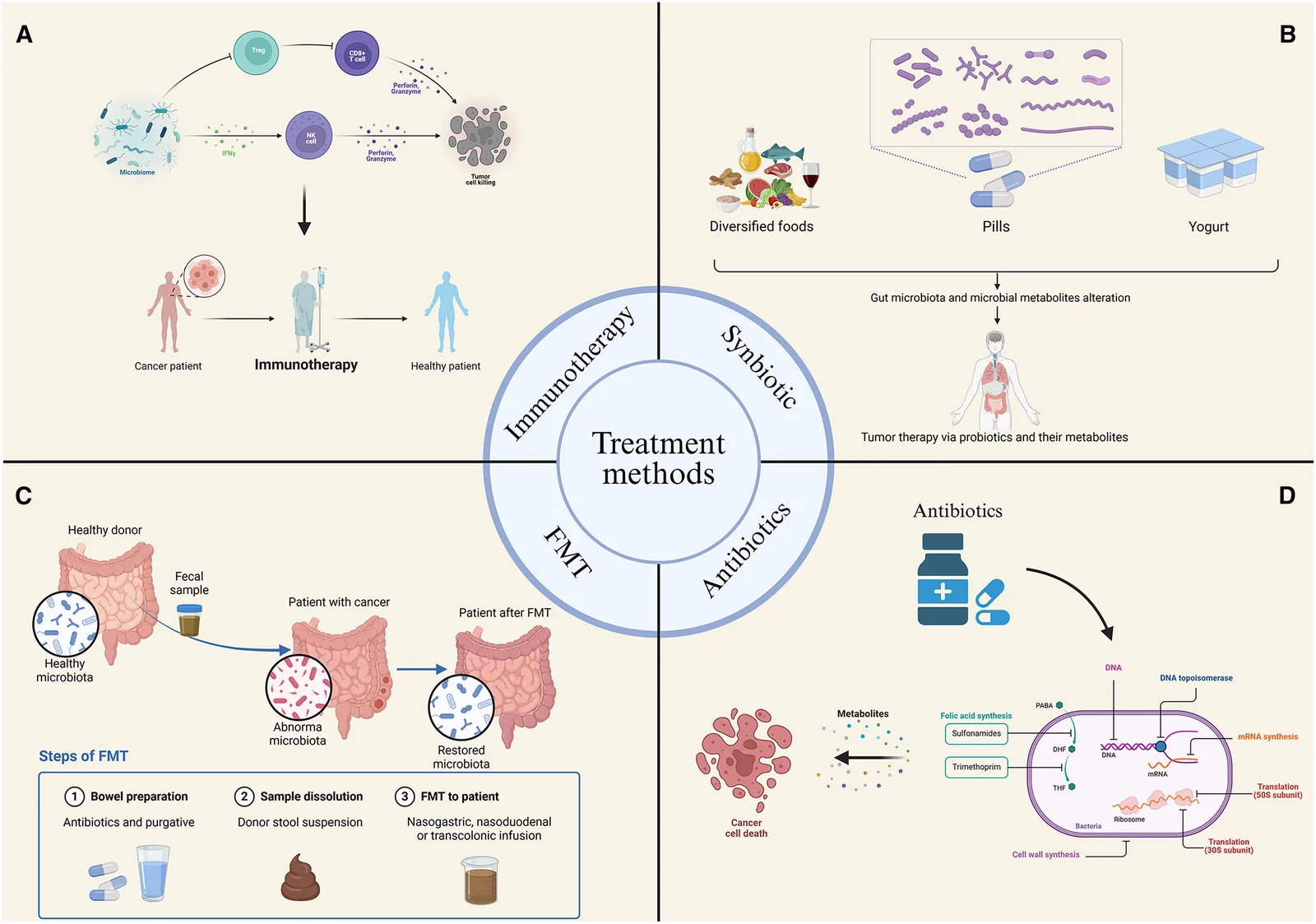
Therapeutic modulation of the microbiome-immunity axis in cancer (A) Immune checkpoint: intratumoral microbes enhance anti-tumor immunity. (B) Synbiotics: combined probiotics + prebiotics reshape microbial and metabolite profiles. (C) FMT: stepwise transfer of healthy-donor microbiota (donor screening → bowel prep → stool suspension → nasoduodenal/transcolonic infusion). (D) Antibiotics: selective depletion of pathobionts to restore microbial equilibrium.

Step three engineers the guards themselves. Synthetic biology-refined live biotherapeutic products (LBPs) bypass the ecological lottery by encoding tumor-specific instructions: hypoxia-, quorum-, or pH-inducible circuits secrete IL-12 or GM-CSF, convert 5-fluorocytosine into local 5-FU, or display soluble TIGIT decoys that adsorb Fusobacterium Fap2 adhesins, all without systemic exposure.^111^^,^^161^^,^^162^^,^^163^ These chassis strains are equipped with genetic circuits that couple tumor-specific environmental cues (hypoxia, quorum signals, or low pH) to the expression of therapeutic cargos. Upon colonization, they can (1) secrete immune-stimulatory cytokines such as IL-12 or GM-CSF, converting an immune-excluded TME into an IFN-γ-rich, dendritic-cell-activated milieu; (2) release pro-drugs, e.g., converting 5-fluorocytosine into 5-FU, directly inside malignant beds, thereby achieving local cytotoxic concentrations while sparing systemic toxicity; or (3) display soluble TIGIT decoys that adsorb *Fusobacterium* Fap2 adhesins, functionally neutralizing a key ITM-mediated checkpoint blockade without antibiotics.^111^^,^^161^ CRISPR-armored *Lactobacillus rhamnosus* GG co-expressing galacto-oligosaccharide enzymes and IL-15 super-agonist peptides persistently colonizes murine CRC, expands resident NK-cell cytotoxicity and reduces post-operative infections more effectively than wild-type probiotics, while kill-switch circuits triggered by exogenous trehalose provide an emergency off-button.^164^^,^^165^^,^^166^^,^^167^ By merging programmable biology with real-time imaging, LBPs convert ITM from a passive biomarker into a programmable delivery system, an advanced component of precision oncology (Figure 6).

Current strategies overly rely on broad-spectrum antibiotics to reduce tumor bacterial load; however, this approach disrupts beneficial commensals and may impair checkpoint inhibitor efficacy, a risk-benefit profile increasingly questioned by the field. We argue that future efforts must prioritize precision interventions: engineered live biotherapeutics designed to release therapeutic payloads specifically within the TME, offering a sophisticated alternative to ecosystem-wide depletion. This conceptual shift from broad-spectrum clearance to targeted functional reprogramming marks the critical transition from generic microbiome modulation to patient-specific therapeutic design.

## Discussion

TME has ceased to be a mere backdrop; it is now recognized as a dynamic ecosystem that fundamentally reshapes cancer biology. This review systematically synthesizes the expanding body of knowledge on ITM in two high-impact malignancies, CRC and lung cancer, offering a unified framework that spans detection methodologies, comparative microbial ecology, mechanistic insights, and translational applications. Placing CRC and lung cancer under the same analytical lens fills a conceptual gap in microbiome-oncology research. Previous reviews have treated each organ separately, limiting insight into systemic microbial drivers. The CRC-lung dyad provides the clearest human model in which the route of microbial spread is anatomically defined, the same pathobiont species are repeatedly recovered, and clinical outcome data are mature. Findings in the gut can forecast pulmonary risk, whereas mechanisms uncovered in the airway, such as hypoxia-driven selection for *Veillonella*, inform how *Fusobacterium* might thrive in inflamed colonic mucosa. This reciprocal illumination transforms a descriptive comparison into a mechanistic framework that justifies targeting conserved microbial effectors in patients with either malignancy. Its core innovations lie in three areas: a rigorous side-by-side comparison of ITM signatures across anatomically distinct cancers, formal elevation of the gut-lung axis from speculative concept to validated driver of CRLM, and a forward-looking integration of spatial-omics, non-bacterial domains, and engineered live biotherapeutics.

Despite originating from different primary reservoirs, gut lumen versus airway epithelium, the ITMs of CRC and lung tumors exhibit striking functional convergence among shared oral-derived taxa. Mechanistically, these microbes promote oncogenesis through a conserved triad: (1) chronic inflammatory signaling via pattern-recognition receptor pathways such as TLR4-NF-κB; (2) active immune evasion, exemplified by *F. nucleatum*’s Fap2-TIGIT axis that disables cytotoxic T and NK cells; and (3) direct genotoxic insults and metabolic reprogramming that foster DNA damage, mutagenesis, and nutrient addiction. The cumulative evidence positions the gut-lung axis as a *bona fide* systemic conduit through which intestinal dysbiosis remotely conditions the pulmonary immune milieu, thereby facilitating metastatic seeding. Notwithstanding these conceptual and experimental advances, several fundamental challenges must be surmounted to translate ITM biology into routine clinical practice.

### Methodological and reproducibility hurdles

The foremost bottleneck facing ITM research is the absence of methodological harmonization. Because ITM samples are vanishingly low in biomass, even femtogram levels of contaminating DNA from reagents, consumables, or laboratory air, the notorious “kitome,” can swamp authentic microbial signals. Divergent protocols for tissue collection, DNA extraction, 16S/ITS variable-region choice, and bioinformatic processing have produced taxonomic profiles that often conflict between centers, undermining meta-analyses and delaying biomarker qualification. To convert intriguing observations into clinically actionable tools, the field must mandate rigorously processed negative and positive controls in every batch, adopt extended minimum reporting guidelines (e.g., STROME-ITM), and participate in international ring trials that benchmark protocols across continents. Only when a microbial signature proves reproducible in different laboratories, with different kits, and on different sequencing platforms will it merit diagnostic or therapeutic deployment.

### Establishing causality and context dependency

A deeper scientific chasm lies in distinguishing causation from correlation. Although pathobionts such as *F. nucleatum* are repeatedly enriched in poor-prognosis tumors, definitive proof that the bacterium drives metastasis, rather than simply exploiting an immunosuppressed niche, requires sophisticated *in vivo* barcoding systems, patient-derived organoid models, and microbe-edited isogenic lines. Moreover, the functional impact of any given ITM component is exquisitely context-dependent. The same SCFA that induces apoptosis in healthy colonic epithelium can serve as an energetic substrate for an established tumor or foster immune tolerance at a metastatic site. Future studies must therefore stratify by tumor subsite, stage, mutational landscape, and host immune genotype to dissect these nuanced, and sometimes paradoxical roles.

### Future directions: Integrating high-resolution data and precision intervention

To transcend current limitations, research efforts must converge on two fronts: spatial resolution and therapeutic precision. Bulk sequencing averages signal across malignant, stromal, and immune compartments, obscuring whether a microbe sits inside a cancer cell, an exhausted CD8^+^ T cell or the extracellular matrix. Emerging multiplexed ion-beam imaging and *in situ* hybridization platforms now overcome this blur by simultaneously visualizing bacterial 16S rRNA, host transcripts and immune-cell phenotypes at single-cell resolution. Correlating microbial proximity to the invasive front, tertiary lymphoid structures or hypoxic niches with local immune activation will finally supply the spatial functional mapping required for rational targeting.

Translational programs must exploit this cartography by graduating from blunt instruments, broad-spectrum antibiotics that eradicate beneficial taxa and fecal microbiota transplantation whose complexity defies quality control, to precision microbiota modulation. Engineered LBPs represent the vanguard of this transition: programmable chassis instructed to scavenge tumor-specific nutrients, secrete PD-1 nanobodies or express soluble TIGIT decoys that neutralize *Fusobacterium* adhesins, all while carrying CRISPR-based kill-switches for immediate safety off-ramps. Coupling such LBPs to real-time imaging or circulating microbial DNA readouts will enable closed-loop, patient-specific manipulation of the ITM-immunity axis, synergizing with immune checkpoint inhibitors and conventional chemotherapies to turn the tumor microbiome into a routinely modifiable arm of precision oncology.

Ultimately, the successful translation of these concepts into routine clinical practice necessitates a shift from cross-sectional discovery to longitudinal validation. Future clinical trials must move beyond static profiling to monitor the dynamic evolution of the ITM throughout the course of disease and treatment. Specifically, elucidating how standard-of-care therapies, such as chemotherapy, radiotherapy, and immunotherapy, selectively remodel the tumor-resident microbial community will be crucial for identifying the optimal therapeutic windows for microbiome-targeted interventions. Furthermore, large-scale, multicenter cohorts are indispensable to distinguish universal microbial drivers from geography-specific commensals, ensuring that emerging biomarkers and therapeutic strategies are robust across diverse patient populations.

From our synthesis, we posit that the ITM should no longer be viewed as a passive bystander but as an active, therapeutically tractable driver of cancer progression. We argue that future efforts must prioritize spatially resolved, longitudinal studies over bulk sequencing snapshots, and that precision microbiome modulation will likely require sequential, patient-specific protocols rather than one-size-fits-all interventions.

## Conclusion

CRC and lung cancers, anatomically remote yet systemically linked, share a discrete ITM dominated by oral commensal bacteria such as *Fusobacterium*, *Streptococcus*, and *Veillonella*. These organisms deliver genotoxins, enforce chronic NF-κB/STAT3 signaling, and disable cytotoxic immunity through TIGIT engagement, thereby accelerating local growth and distal metastasis via the gut-lung axis. Spatial multi-omics localizes each microbe to specific tumor niches, while non-invasive fecal or salivary signatures are being explored for early detection and prediction of immunotherapy resistance. Therapeutically, narrow-spectrum antibiotics, rigorously screened fecal microbiota transplantation, and programmable live biotherapeutics provide a graded arsenal that can silence pathogens, reinstall immune-supportive taxa, or deliver oncolytic payloads directly within the tumor bed. Standardizing contamination-aware protocols, validating causality in prospective cohorts, and embedding kill-switch safety circuits into engineered strains remain the critical next steps to convert the ITM from a biological curiosity into a routinely modifiable arm of precision oncology.

## Ethics approval and consent to participate

Not applicable.

## Consent for publication

All authors have read and approved the final manuscript.

## Acknowledgments

This work was supported by the General Project of Liaoning Provincial Department of Education (JYTMS20230566 to Y.L.) and the 10.13039/501100005047Natural Science Foundation of Liaoning Province (2019-ZD-0648 to Y.L. and 2019-ZD-0622 to D.W.).

## Author contributions

Y.L., D.W., and Y.W. conceived the study; J.L., X.D., and W.L. collected the data; Y.W. and J.L. drafted the manuscript and prepared the figures; Y.L. and D.W. revised the manuscript.

## Declaration of interests

The authors declare that they have no conflicts of interest.

## References

[1] Wu S., Powers S., Zhu W., Hannun Y.A. (2016). Substantial contribution of extrinsic risk factors to cancer development. Nature.

[2] Bano Y., Shrivastava A., Shukla P., Chaudhary A.A., Khan S.U.D., Khan S. (2025). The implication of microbiome in lungs cancer: mechanisms and strategies of cancer growth, diagnosis and therapy. Crit. Rev. Microbiol..

[3] Yuan S., Almagro J., Fuchs E. (2024). Beyond genetics: driving cancer with the tumour microenvironment behind the wheel. Nat. Rev. Cancer.

[4] Torres M.K.d.S., Pereira Neto G.D.S., Cayres Vallinoto I.M.V., Reis L.O., Vallinoto A.C.R. (2025). The Impact of Oncogenic Viruses on Cancer Development: A Narrative Review. Biology.

[5] Khan S., Imran A., Khan A.A., Abul Kalam M., Alshamsan A. (2016). Systems Biology Approaches for the Prediction of Possible Role of Chlamydia pneumoniae Proteins in the Etiology of Lung Cancer. PLoS One.

[6] Wang Y., Imran A., Shami A., Chaudhary A.A., Khan S. (2021). Decipher the Helicobacter pylori Protein Targeting in the Nucleus of Host Cell and their Implications in Gallbladder Cancer: An insilico approach. J. Cancer.

[7] Khan S., Zaidi S., Alouffi A.S., Hassan I., Imran A., Khan R.A. (2020). Computational Proteome-Wide Study for the Prediction of Escherichia coli Protein Targeting in Host Cell Organelles and Their Implication in Development of Colon Cancer. ACS Omega.

[8] Khan S., Zakariah M., Rolfo C., Robrecht L., Palaniappan S. (2017). Prediction of mycoplasma hominis proteins targeting in mitochondria and cytoplasm of host cells and their implication in prostate cancer etiology. Oncotarget.

[9] Siegel R.L., Miller K.D., Fuchs H.E., Jemal A. (2022). Cancer statistics, 2022. CA Cancer J. Clin..

[10] Bray F., Ferlay J., Soerjomataram I., Siegel R.L., Torre L.A., Jemal A. (2018). Global cancer statistics 2018: GLOBOCAN estimates of incidence and mortality worldwide for 36 cancers in 185 countries. CA Cancer J. Clin..

[11] Thai A.A., Solomon B.J., Sequist L.V., Gainor J.F., Heist R.S. (2021). Lung cancer. Lancet.

[12] Spill F., Reynolds D.S., Kamm R.D., Zaman M.H. (2016). Impact of the physical microenvironment on tumor progression and metastasis. Curr. Opin. Biotechnol..

[13] Del Prete A., Schioppa T., Tiberio L., Stabile H., Sozzani S. (2017). Leukocyte trafficking in tumor microenvironment. Curr. Opin. Pharmacol..

[14] Cao Y., Xia H., Tan X., Shi C., Ma Y., Meng D., Zhou M., Lv Z., Wang S., Jin Y. (2024). Intratumoural microbiota: a new frontier in cancer development and therapy. Signal Transduct. Target. Ther..

[15] Arneth B. (2019). Tumor Microenvironment. Medicina (Kaunas, Lithuania).

[16] Yang L., Li A., Wang Y., Zhang Y. (2023). Intratumoral microbiota: roles in cancer initiation, development and therapeutic efficacy. Signal Transduct. Target. Ther..

[17] Mouradov D., Greenfield P., Li S., In E.J., Storey C., Sakthianandeswaren A., Georgeson P., Buchanan D.D., Ward R.L., Hawkins N.J. (2023). Oncomicrobial Community Profiling Identifies Clinicomolecular and Prognostic Subtypes of Colorectal Cancer. Gastroenterology.

[18] Ferrari V., Rescigno M. (2023). The intratumoral microbiota: friend or foe?. Trends Cancer.

[19] Galeano Niño J.L., Wu H., LaCourse K.D., Kempchinsky A.G., Baryiames A., Barber B., Futran N., Houlton J., Sather C., Sicinska E. (2022). Effect of the intratumoral microbiota on spatial and cellular heterogeneity in cancer. Nature.

[20] Budden K.F., Gellatly S.L., Wood D.L.A., Cooper M.A., Morrison M., Hugenholtz P., Hansbro P.M. (2017). Emerging pathogenic links between microbiota and the gut-lung axis. Nat. Rev. Microbiol..

[21] Riihimäki M., Hemminki A., Sundquist J., Hemminki K. (2016). Patterns of metastasis in colon and rectal cancer. Sci. Rep..

[22] Moorcraft S.Y., Ladas G., Bowcock A., Chau I. (2016). Management of resectable colorectal lung metastases. Clin. Exp. Metastasis.

[23] Wang N., Fang J.Y. (2023). Fusobacterium nucleatum, a key pathogenic factor and microbial biomarker for colorectal cancer. Trends Microbiol..

[24] Younginger B.S., Mayba O., Reeder J., Nagarkar D.R., Modrusan Z., Albert M.L., Byrd A.L. (2023). Enrichment of oral-derived bacteria in inflamed colorectal tumors and distinct associations of Fusobacterium in the mesenchymal subtype. Cell Rep. Med..

[25] Lin Y., Lau H.C.H., Liu C., Ding X., Sun Y., Rong J., Zhang X., Wang L., Yuan K., Miao Y. (2025). Multi-cohort analysis reveals colorectal cancer tumor location-associated fecal microbiota and their clinical impact. Cell Host Microbe.

[26] Cardona Zorrilla A.F. (2025). Exploring the role of the oral microbiome in saliva, sputum, bronchoalveolar fluid, and tissue tumor in lung cancer: A systematic review. J. Clin. Oncol..

[27] Zhou M., Liu Y., Yin X., Gong J., Li J. (2025). The role of oral microbiota in lung carcinogenesis through the oral-lung axis: a comprehensive review of mechanisms and therapeutic potential. Discov. Oncol..

[28] Overmann J., Abt B., Sikorski J. (2017). Present and Future of Culturing Bacteria. Annu. Rev. Microbiol..

[29] Chen J., Li T., Ye C., Zhong J., Huang J.D., Ke Y., Sun H. (2023). The Lung Microbiome: A New Frontier for Lung and Brain Disease. Int. J. Mol. Sci..

[30] Castellarin M., Warren R.L., Freeman J.D., Dreolini L., Krzywinski M., Strauss J., Barnes R., Watson P., Allen-Vercoe E., Moore R.A., Holt R.A. (2012). Fusobacterium nucleatum infection is prevalent in human colorectal carcinoma. Genome Res..

[31] Klindworth A., Pruesse E., Schweer T., Peplies J., Quast C., Horn M., Glöckner F.O. (2013). Evaluation of general 16S ribosomal RNA gene PCR primers for classical and next-generation sequencing-based diversity studies. Nucleic Acids Res..

[32] Fida M., Wolf M.J., Hamdi A., Vijayvargiya P., Esquer Garrigos Z., Khalil S., Greenwood-Quaintance K.E., Thoendel M.J., Patel R. (2021). Detection of Pathogenic Bacteria From Septic Patients Using 16S Ribosomal RNA Gene-Targeted Metagenomic Sequencing. Clin. Infect. Dis..

[33] Purushothaman S., Meola M., Egli A. (2022). Combination of Whole Genome Sequencing and Metagenomics for Microbiological Diagnostics. Int. J. Mol. Sci..

[34] Zeller G., Tap J., Voigt A.Y., Sunagawa S., Kultima J.R., Costea P.I., Amiot A., Böhm J., Brunetti F., Habermann N. (2014). Potential of fecal microbiota for early-stage detection of colorectal cancer. Mol. Syst. Biol..

[35] Shi Y., Wang G., Lau H.C.H., Yu J. (2022). Metagenomic Sequencing for Microbial DNA in Human Samples: Emerging Technological Advances. Int. J. Mol. Sci..

[36] Mima K., Sukawa Y., Nishihara R., Qian Z.R., Yamauchi M., Inamura K., Kim S.A., Masuda A., Nowak J.A., Nosho K. (2015). Fusobacterium nucleatum and T Cells in Colorectal Carcinoma. JAMA Oncol..

[37] Nejman D., Livyatan I., Fuks G., Gavert N., Zwang Y., Geller L.T., Rotter-Maskowitz A., Weiser R., Mallel G., Gigi E. (2020). The human tumor microbiome is composed of tumor type-specific intracellular bacteria. Science (New York, N.Y.).

[38] Aznab M., Izadi B., Amirian F., Khazaei S., Madani S.H., Ramezani M. (2022). Comparison of Immunohistochemical Methods (IHC) and Fluorescent in Situ Hybridization (FISH) in the Detection of HER 2/Neu Gene in Kurdish Patients with Breast Cancer in Western Iran. Int. J. Hematol. Oncol. Stem Cell Res..

[39] Liu X., Song Y., Li R. (2021). The use of combined PCR, fluorescence in situ hybridisation and immunohistochemical staining to diagnose mucormycosis from formalin-fixed paraffin-embedded tissues. Mycoses.

[40] Prudent E., Raoult D. (2019). Fluorescence in situ hybridization, a complementary molecular tool for the clinical diagnosis of infectious diseases by intracellular and fastidious bacteria. FEMS Microbiol. Rev..

[41] Amann R., Fuchs B.M. (2008). Single-cell identification in microbial communities by improved fluorescence in situ hybridization techniques. Nat. Rev. Microbiol..

[42] Chia M., Pop M., Salzberg S.L., Nagarajan N. (2025). Challenges and Opportunities in Analyzing Cancer-Associated Microbiomes. Cancer Res..

[43] Jang S., Lee E.J., Park S., Lim H., Ahn B., Huh Y., Koh H., Park Y.R. (2025). Spatial host-microbiome profiling demonstrates bacterial-associated host transcriptional alterations in pediatric ileal Crohn's disease. Microbiome.

[44] Wong-Rolle A., Dong Q., Zhu Y., Divakar P., Hor J.L., Kedei N., Wong M., Tillo D., Conner E.A., Rajan A. (2022). Spatial meta-transcriptomics reveal associations of intratumor bacteria burden with lung cancer cells showing a distinct oncogenic signature. J. Immunother. Cancer.

[45] Barbosa A., Miranda S., Azevedo N.F., Cerqueira L., Azevedo A.S. (2023). Imaging biofilms using fluorescence in situ hybridization: seeing is believing. Front. Cell. Infect. Microbiol..

[46] Shi H., Shi Q., Grodner B., Lenz J.S., Zipfel W.R., Brito I.L., De Vlaminck I. (2020). Highly multiplexed spatial mapping of microbial communities. Nature.

[47] Lu Y.Q., Qiao H., Tan X.R., Liu N. (2024). Broadening oncological boundaries: the intratumoral microbiota. Trends Microbiol..

[48] Galeano Niño J.L., Wu H., LaCourse K.D., Srinivasan H., Fitzgibbon M., Minot S.S., Sather C., Johnston C.D., Bullman S. (2023). INVADEseq to identify cell-adherent or invasive bacteria and the associated host transcriptome at single-cell-level resolution. Nat. Protoc..

[49] Stinson L.F., Keelan J.A., Payne M.S. (2019). Identification and removal of contaminating microbial DNA from PCR reagents: impact on low-biomass microbiome analyses. Lett. Appl. Microbiol..

[50] Lagier J.C., Edouard S., Pagnier I., Mediannikov O., Drancourt M., Raoult D. (2015). Current and past strategies for bacterial culture in clinical microbiology. Clin. Microbiol. Rev..

[51] Kapinusova G., Lopez Marin M.A., Uhlik O. (2023). Reaching unreachables: Obstacles and successes of microbial cultivation and their reasons. Front. Microbiol..

[52] Yan C., Owen J.S., Seo E.Y., Jung D., He S. (2023). Microbial Interaction is Among the Key Factors for Isolation of Previous Uncultured Microbes. J. Microbiol..

[53] Wan X., Yang Q., Wang X., Bai Y., Liu Z. (2023). Isolation and Cultivation of Human Gut Microorganisms: A Review. Microorganisms.

[54] Lane D.J., Pace B., Olsen G.J., Stahl D.A., Sogin M.L., Pace N.R. (1985). Rapid determination of 16S ribosomal RNA sequences for phylogenetic analyses. Proc. Natl. Acad. Sci. USA.

[55] Church D.L., Cerutti L., Gürtler A., Griener T., Zelazny A., Emler S. (2020). Performance and Application of 16S rRNA Gene Cycle Sequencing for Routine Identification of Bacteria in the Clinical Microbiology Laboratory. Clin. Microbiol. Rev..

[56] Janda J.M., Abbott S.L. (2007). 16S rRNA gene sequencing for bacterial identification in the diagnostic laboratory: pluses, perils, and pitfalls. J. Clin. Microbiol..

[57] Osman M.A., Neoh H.M., Ab Mutalib N.S., Chin S.F., Jamal R. (2018). 16S rRNA Gene Sequencing for Deciphering the Colorectal Cancer Gut Microbiome: Current Protocols and Workflows. Front. Microbiol..

[58] Tringe S.G., Rubin E.M. (2005). Metagenomics: DNA sequencing of environmental samples. Nat. Rev. Genet..

[59] Schloss P.D., Handelsman J. (2005). Metagenomics for studying unculturable microorganisms: cutting the Gordian knot. Genome Biol..

[60] Liu Y.X., Qin Y., Chen T., Lu M., Qian X., Guo X., Bai Y. (2021). A practical guide to amplicon and metagenomic analysis of microbiome data. Protein Cell.

[61] Tan W.C.C., Nerurkar S.N., Cai H.Y., Ng H.H.M., Wu D., Wee Y.T.F., Lim J.C.T., Yeong J., Lim T.K.H. (2020). Overview of multiplex immunohistochemistry/immunofluorescence techniques in the era of cancer immunotherapy. Cancer Commun..

[62] Harms P.W., Frankel T.L., Moutafi M., Rao A., Rimm D.L., Taube J.M., Thomas D., Chan M.P., Pantanowitz L. (2023). Multiplex Immunohistochemistry and Immunofluorescence: A Practical Update for Pathologists. Mod. Pathol..

[63] Magaki S., Hojat S.A., Wei B., So A., Yong W.H. (2019). An Introduction to the Performance of Immunohistochemistry. Methods Mol. Biol..

[64] Frickmann H., Zautner A.E., Moter A., Kikhney J., Hagen R.M., Stender H., Poppert S. (2017). Fluorescence in situ hybridization (FISH) in the microbiological diagnostic routine laboratory: a review. Crit. Rev. Microbiol..

[65] Kempf V.A.J., Trebesius K., Autenrieth I.B. (2000). Fluorescent In situ hybridization allows rapid identification of microorganisms in blood cultures. J. Clin. Microbiol..

[66] Kostic A.D., Gevers D., Pedamallu C.S., Michaud M., Duke F., Earl A.M., Ojesina A.I., Jung J., Bass A.J., Tabernero J. (2012). Genomic analysis identifies association of Fusobacterium with colorectal carcinoma. Genome Res..

[67] Emens L.A., Romero P.J., Anderson A.C., Bruno T.C., Capitini C.M., Collyar D., Gulley J.L., Hwu P., Posey A.D., Silk A.W., Wargo J.A. (2024). Challenges and opportunities in cancer immunotherapy: a Society for Immunotherapy of Cancer (SITC) strategic vision. J. Immunother. Cancer.

[68] Galeano Niño J.L., Ponath F., Ajisafe V.A., Becker C.R., Kempchinsky A.G., Zepeda-Rivera M.A., Gomez J.A., Wu H., Terrazas J.G., Bouzek H. (2026). Tumor-infiltrating bacteria disrupt cancer epithelial cell interactions and induce cell-cycle arrest. Cancer Cell.

[69] Long J., Wang J., Xiao C., You F., Jiang Y., Li X. (2024). Intratumoral microbiota in colorectal cancer: focus on specific distribution and potential mechanisms. Cell Commun. Signal..

[70] Wang M., Yu F., Li P. (2023). Intratumor microbiota in cancer pathogenesis and immunity: from mechanisms of action to therapeutic opportunities. Front. Immunol..

[71] Souza V.G.P., Forder A., Pewarchuk M.E., Telkar N., de Araujo R.P., Stewart G.L., Vieira J., Reis P.P., Lam W.L. (2023). The Complex Role of the Microbiome in Non-Small Cell Lung Cancer Development and Progression. Cells.

[72] Zhao L., Cho W.C., Nicolls M.R. (2021). Colorectal Cancer-Associated Microbiome Patterns and Signatures. Front. Genet..

[73] Zhu H., Li M., Bi D., Yang H., Gao Y., Song F., Zheng J., Xie R., Zhang Y., Liu H. (2024). Fusobacterium nucleatum promotes tumor progression in KRAS p.G12D-mutant colorectal cancer by binding to DHX15. Nat. Commun..

[74] Mima K., Nishihara R., Qian Z.R., Cao Y., Sukawa Y., Nowak J.A., Yang J., Dou R., Masugi Y., Song M. (2016). Fusobacterium nucleatum in colorectal carcinoma tissue and patient prognosis. Gut.

[75] Dai Z., Coker O.O., Nakatsu G., Wu W.K.K., Zhao L., Chen Z., Chan F.K.L., Kristiansen K., Sung J.J.Y., Wong S.H., Yu J. (2018). Multi-cohort analysis of colorectal cancer metagenome identified altered bacteria across populations and universal bacterial markers. Microbiome.

[76] Jiang M., Yang Z., Dai J., Wu T., Jiao Z., Yu Y., Ning K., Chen W., Yang A. (2023). Intratumor microbiome: selective colonization in the tumor microenvironment and a vital regulator of tumor biology. MedComm.

[77] Jans C., Boleij A. (2018). The Road to Infection: Host-Microbe Interactions Defining the Pathogenicity of Streptococcus bovis/Streptococcus equinus Complex Members. Front. Microbiol..

[78] Qu R., Zhang Y., Ma Y., Zhou X., Sun L., Jiang C., Zhang Z., Fu W. (2023). Role of the Gut Microbiota and Its Metabolites in Tumorigenesis or Development of Colorectal Cancer. Adv. Sci..

[79] Yu G., Gail M.H., Consonni D., Carugno M., Humphrys M., Pesatori A.C., Caporaso N.E., Goedert J.J., Ravel J., Landi M.T. (2016). Characterizing human lung tissue microbiota and its relationship to epidemiological and clinical features. Genome Biol..

[80] Zeng W., Zhao C., Yu M., Chen H., Pan Y., Wang Y., Bao H., Ma H., Ma S. (2022). Alterations of lung microbiota in patients with non-small cell lung cancer. Bioengineered.

[81] Lucaciu S.R., Domokos B., Puiu R., Ruta V., Motoc S.N., Rajnoveanu R., Todea D., Stoia A.M., Man A.M. (2024). Lung Microbiome in Lung Cancer: A Systematic Review. Microorganisms.

[82] Huang D., Su X., Yuan M., Zhang S., He J., Deng Q., Qiu W., Dong H., Cai S. (2019). The characterization of lung microbiome in lung cancer patients with different clinicopathology. Am. J. Cancer Res..

[83] Hosgood H.D., Sapkota A.R., Rothman N., Rohan T., Hu W., Xu J., Vermeulen R., He X., White J.R., Wu G. (2014). The potential role of lung microbiota in lung cancer attributed to household coal burning exposures. Environ. Mol. Mutagen..

[84] Zheng Y., Fang Z., Xue Y., Zhang J., Zhu J., Gao R., Yao S., Ye Y., Wang S., Lin C. (2020). Specific gut microbiome signature predicts the early-stage lung cancer. Gut Microbes.

[85] Wong-Rolle A., Wei H.K., Zhao C., Jin C. (2021). Unexpected guests in the tumor microenvironment: microbiome in cancer. Protein Cell.

[86] Gomes S., Cavadas B., Ferreira J.C., Marques P.I., Monteiro C., Sucena M., Sousa C., Vaz Rodrigues L., Teixeira G., Pinto P. (2019). Profiling of lung microbiota discloses differences in adenocarcinoma and squamous cell carcinoma. Sci. Rep..

[87] Baranova E., Druzhinin V., Matskova L., Demenkov P., Volobaev V., Minina V., Larionov A., Titov V. (2022). Sputum Microbiome Composition in Patients with Squamous Cell Lung Carcinoma. Life.

[88] Liu W., Zhang X., Xu H., Li S., Lau H.C.H., Chen Q., Zhang B., Zhao L., Chen H., Sung J.J.Y., Yu J. (2021). Microbial Community Heterogeneity Within Colorectal Neoplasia and its Correlation With Colorectal Carcinogenesis. Gastroenterology.

[89] Wang Y., Wang Y., Zhou Y., Feng Y., Sun T., Xu J. (2024). Tumor-related fungi and crosstalk with gut fungi in the tumor microenvironment. Cancer Biol. Med..

[90] Gao R., Xia K., Wu M., Zhong H., Sun J., Zhu Y., Huang L., Wu X., Yin L., Yang R. (2022). Alterations of Gut Mycobiota Profiles in Adenoma and Colorectal Cancer. Front. Cell. Infect. Microbiol..

[91] Coker O.O., Nakatsu G., Dai R.Z., Wu W.K.K., Wong S.H., Ng S.C., Chan F.K.L., Sung J.J.Y., Yu J. (2019). Enteric fungal microbiota dysbiosis and ecological alterations in colorectal cancer. Gut.

[92] Yang Q., Ouyang J., Pi D., Feng L., Yang J. (2022). Malassezia in Inflammatory Bowel Disease: Accomplice of Evoking Tumorigenesis. Front. Immunol..

[93] Schwabe R.F., Jobin C. (2013). The microbiome and cancer. Nat. Rev. Cancer.

[94] Dang A.T., Marsland B.J. (2019). Microbes, metabolites, and the gut-lung axis. Mucosal Immunol..

[95] Tang W., Li F., Zheng H., Zhou S., Li C., Xu X., Fu J. (2025). Unveiling hidden players: the role of intratumoral microbiota in gastrointestinal cancer dynamics. J. Cancer Res. Clin. Oncol..

[96] Fan Y., Mao R., Yang J. (2013). NF-κB and STAT3 signaling pathways collaboratively link inflammation to cancer. Protein Cell.

[97] Goodwin A.C., Destefano Shields C.E., Wu S., Huso D.L., Wu X., Murray-Stewart T.R., Hacker-Prietz A., Rabizadeh S., Woster P.M., Sears C.L., Casero R.A. (2011). Polyamine catabolism contributes to enterotoxigenic Bacteroides fragilis-induced colon tumorigenesis. Proc. Natl. Acad. Sci. USA.

[98] Dejea C.M., Fathi P., Craig J.M., Boleij A., Taddese R., Geis A.L., Wu X., DeStefano Shields C.E., Hechenbleikner E.M., Huso D.L. (2018). Patients with familial adenomatous polyposis harbor colonic biofilms containing tumorigenic bacteria. Science (New York, N.Y.).

[99] Chung L., Thiele Orberg E., Geis A.L., Chan J.L., Fu K., DeStefano Shields C.E., Dejea C.M., Fathi P., Chen J., Finard B.B. (2018). Bacteroides fragilis Toxin Coordinates a Pro-carcinogenic Inflammatory Cascade via Targeting of Colonic Epithelial Cells. Cell Host Microbe.

[100] Zhou Z., Chen J., Yao H., Hu H. (2018). Fusobacterium and Colorectal Cancer. Front. Oncol..

[101] Gao Z., Guo B., Gao R., Zhu Q., Qin H. (2015). Microbiota disbiosis is associated with colorectal cancer. Front. Microbiol..

[102] Chen T., Li Q., Wu J., Wu Y., Peng W., Li H., Wang J., Tang X., Peng Y., Fu X. (2018). Fusobacterium nucleatum promotes M2 polarization of macrophages in the microenvironment of colorectal tumours via a TLR4-dependent mechanism. Cancer Immunol. Immunother..

[103] Yamamoto S., Kinugasa H., Hirai M., Terasawa H., Yasutomi E., Oka S., Ohmori M., Yamasaki Y., Inokuchi T., Harada K. (2021). Heterogeneous distribution of Fusobacterium nucleatum in the progression of colorectal cancer. J. Gastroenterol. Hepatol..

[104] Guo P., Tian Z., Kong X., Yang L., Shan X., Dong B., Ding X., Jing X., Jiang C., Jiang N., Yu Y. (2020). FadA promotes DNA damage and progression of Fusobacterium nucleatum-induced colorectal cancer through up-regulation of chk2. J. Exp. Clin. Cancer Res..

[105] Park H.E., Kim J.H., Cho N.Y., Lee H.S., Kang G.H. (2017). Intratumoral Fusobacterium nucleatum abundance correlates with macrophage infiltration and CDKN2A methylation in microsatellite-unstable colorectal carcinoma. Virchows Arch..

[106] Hong J., Guo F., Lu S.Y., Shen C., Ma D., Zhang X., Xie Y., Yan T., Yu T., Sun T. (2021). F. nucleatum targets lncRNA ENO1-IT1 to promote glycolysis and oncogenesis in colorectal cancer. Gut.

[107] Xia X., Wu W.K.K., Wong S.H., Liu D., Kwong T.N.Y., Nakatsu G., Yan P.S., Chuang Y.M., Chan M.W.Y., Coker O.O. (2020). Bacteria pathogens drive host colonic epithelial cell promoter hypermethylation of tumor suppressor genes in colorectal cancer. Microbiome.

[108] Koi M., Okita Y., Carethers J.M. (2018). Fusobacterium nucleatum Infection in Colorectal Cancer: Linking Inflammation, DNA Mismatch Repair and Genetic and Epigenetic Alterations. J. Anus Rectum Colon.

[109] Tang B., Wang K., Jia Y.P., Zhu P., Fang Y., Zhang Z.J., Mao X.H., Li Q., Zeng D.Z. (2016). Fusobacterium nucleatum-Induced Impairment of Autophagic Flux Enhances the Expression of Proinflammatory Cytokines via ROS in Caco-2 Cells. PLoS One.

[110] Borowsky J., Haruki K., Lau M.C., Dias Costa A., Väyrynen J.P., Ugai T., Arima K., da Silva A., Felt K.D., Zhao M. (2021). Association of Fusobacterium nucleatum with Specific T-cell Subsets in the Colorectal Carcinoma Microenvironment. Clin. Cancer Res..

[111] Gur C., Ibrahim Y., Isaacson B., Yamin R., Abed J., Gamliel M., Enk J., Bar-On Y., Stanietsky-Kaynan N., Coppenhagen-Glazer S. (2015). Binding of the Fap2 protein of Fusobacterium nucleatum to human inhibitory receptor TIGIT protects tumors from immune cell attack. Immunity.

[112] Gur C., Maalouf N., Shhadeh A., Berhani O., Singer B.B., Bachrach G., Mandelboim O. (2019). Fusobacterium nucleatum supresses anti-tumor immunity by activating CEACAM1. Oncoimmunology.

[113] Wu Y., Guo S., Chen F., Li Y., Huang Y., Liu W., Zhang G. (2023). Fn-Dps, a novel virulence factor of Fusobacterium nucleatum, disrupts erythrocytes and promotes metastasis in colorectal cancer. PLoS Pathog..

[114] Zhang Y., Zhang L., Zheng S., Li M., Xu C., Jia D., Qi Y., Hou T., Wang L., Wang B. (2022). Fusobacterium nucleatum promotes colorectal cancer cells adhesion to endothelial cells and facilitates extravasation and metastasis by inducing ALPK1/NF-κB/ICAM1 axis. Gut Microbes.

[115] Kneis B., Wirtz S., Weber K., Denz A., Gittler M., Geppert C., Brunner M., Krautz C., Siebenhüner A.R., Schierwagen R. (2023). Colon Cancer Microbiome Landscaping: Differences in Right- and Left-Sided Colon Cancer and a Tumor Microbiome-Ileal Microbiome Association. Int. J. Mol. Sci..

[116] Bertocchi A., Carloni S., Ravenda P.S., Bertalot G., Spadoni I., Lo Cascio A., Gandini S., Lizier M., Braga D., Asnicar F. (2021). Gut vascular barrier impairment leads to intestinal bacteria dissemination and colorectal cancer metastasis to liver. Cancer Cell.

[117] Maddocks O.D.K., Scanlon K.M., Donnenberg M.S. (2013). An Escherichia coli effector protein promotes host mutation via depletion of DNA mismatch repair proteins. mBio.

[118] Triner D., Devenport S.N., Ramakrishnan S.K., Ma X., Frieler R.A., Greenson J.K., Inohara N., Nunez G., Colacino J.A., Mortensen R.M., Shah Y.M. (2019). Neutrophils Restrict Tumor-Associated Microbiota to Reduce Growth and Invasion of Colon Tumors in Mice. Gastroenterology.

[119] Dohlman A.B., Klug J., Mesko M., Gao I.H., Lipkin S.M., Shen X., Iliev I.D. (2022). A pan-cancer mycobiome analysis reveals fungal involvement in gastrointestinal and lung tumors. Cell.

[120] Mao Q., Ma W., Wang Z., Liang Y., Zhang T., Yang Y., Xia W., Jiang F., Hu J., Xu L. (2020). Differential flora in the microenvironment of lung tumor and paired adjacent normal tissues. Carcinogenesis.

[121] Narunsky-Haziza L., Sepich-Poore G.D., Livyatan I., Asraf O., Martino C., Nejman D., Gavert N., Stajich J.E., Amit G., González A. (2022). Pan-cancer analyses reveal cancer-type-specific fungal ecologies and bacteriome interactions. Cell.

[122] Greathouse K.L., White J.R., Vargas A.J., Bliskovsky V.V., Beck J.A., von Muhlinen N., Polley E.C., Bowman E.D., Khan M.A., Robles A.I. (2018). Interaction between the microbiome and TP53 in human lung cancer. Genome Biol..

[123] Apopa P.L., Alley L., Penney R.B., Arnaoutakis K., Steliga M.A., Jeffus S., Bircan E., Gopalan B., Jin J., Patumcharoenpol P. (2018). PARP1 Is Up-Regulated in Non-small Cell Lung Cancer Tissues in the Presence of the Cyanobacterial Toxin Microcystin. Front. Microbiol..

[124] Lee S.H., Sung J.Y., Yong D., Chun J., Kim S.Y., Song J.H., Chung K.S., Kim E.Y., Jung J.Y., Kang Y.A. (2016). Characterization of microbiome in bronchoalveolar lavage fluid of patients with lung cancer comparing with benign mass like lesions. Lung cancer (Amsterdam, Netherlands).

[125] Zheng L., Xu J., Sai B., Zhu Y., Wang L., Yin N., Yu F., Zhou W., Wu M., Tang J., Xiang J. (2020). Microbiome Related Cytotoxically Active CD8+ TIL Are Inversely Associated With Lung Cancer Development. Front. Oncol..

[126] Jungnickel C., Schmidt L.H., Bittigkoffer L., Wolf L., Wolf A., Ritzmann F., Kamyschnikow A., Herr C., Menger M.D., Spieker T. (2017). IL-17C mediates the recruitment of tumor-associated neutrophils and lung tumor growth. Oncogene.

[127] Guo S., Chen J., Chen F., Zeng Q., Liu W.L., Zhang G. (2020). Exosomes derived from Fusobacterium nucleatum-infected colorectal cancer cells facilitate tumour metastasis by selectively carrying miR-1246/92b-3p/27a-3p and CXCL16. Gut.

[128] Zhang M., Wang Y., Yu L., Zhang Y., Wang Y., Shang Z., Xin Y., Li X., Ning N., Zhang Y., Zhang X. (2023). Fusobacterium nucleatum promotes colorectal cancer metastasis by excretion of miR-122-5p from cells via exosomes. iScience.

[129] Bullman S., Pedamallu C.S., Sicinska E., Clancy T.E., Zhang X., Cai D., Neuberg D., Huang K., Guevara F., Nelson T. (2017). Analysis of Fusobacterium persistence and antibiotic response in colorectal cancer. Science (New York, N.Y.).

[130] McCormack P.M., Burt M.E., Bains M.S., Martini N., Rusch V.W., Ginsberg R.J. (1992). Lung resection for colorectal metastases. 10-year results. Arch. Surg..

[131] Sharma M., Rameshbabu C.S. (2012). Collateral pathways in portal hypertension. J. Clin. Exp. Hepatol..

[132] Inoue A., Sheedy S.P., Heiken J.P., Mohammadinejad P., Graham R.P., Lee H.E., Kelley S.R., Hansel S.L., Bruining D.H., Fidler J.L., Fletcher J.G. (2021). MRI-detected extramural venous invasion of rectal cancer: Multimodality performance and implications at baseline imaging and after neoadjuvant therapy. Insights Imaging.

[133] Liu J., Luo F., Wen L., Zhao Z., Sun H. (2023). Current Understanding of Microbiomes in Cancer Metastasis. Cancers.

[134] Chen S., Su T., Zhang Y., Lee A., He J., Ge Q., Wang L., Si J., Zhuo W., Wang L. (2020). Fusobacterium nucleatum promotes colorectal cancer metastasis by modulating KRT7-AS/KRT7. Gut Microbes.

[135] Zhang Z., Liao Y., Tang D. (2022). Intratumoral microbiota: new frontiers in tumor immunity. Carcinogenesis.

[136] Boleij A., Hechenbleikner E.M., Goodwin A.C., Badani R., Stein E.M., Lazarev M.G., Ellis B., Carroll K.C., Albesiano E., Wick E.C. (2015). The Bacteroides fragilis toxin gene is prevalent in the colon mucosa of colorectal cancer patients. Clin. Infect. Dis..

[137] Rubinstein M.R., Wang X., Liu W., Hao Y., Cai G., Han Y.W. (2013). Fusobacterium nucleatum promotes colorectal carcinogenesis by modulating E-cadherin/β-catenin signaling via its FadA adhesin. Cell Host Microbe.

[138] Chen Y., Peng Y., Yu J., Chen T., Wu Y., Shi L., Li Q., Wu J., Fu X. (2017). Invasive Fusobacterium nucleatum activates beta-catenin signaling in colorectal cancer via a TLR4/P-PAK1 cascade. Oncotarget.

[139] Castro P.R., Bittencourt L.F.F., Larochelle S., Andrade S.P., Mackay C.R., Slevin M., Moulin V.J., Barcelos L.S. (2021). GPR43 regulates sodium butyrate-induced angiogenesis and matrix remodeling. Am. J. Physiol. Heart Circ. Physiol..

[140] He J., Li H., Jia J., Liu Y., Zhang N., Wang R., Qu W., Liu Y., Jia L. (2023). Mechanisms by which the intestinal microbiota affects gastrointestinal tumours and therapeutic effects. Mol. Biomed..

[141] Shalapour S., Karin M. (2020). Cruel to Be Kind: Epithelial, Microbial, and Immune Cell Interactions in Gastrointestinal Cancers. Annu. Rev. Immunol..

[142] Huang J., Huang J. (2022). Microbial Biomarkers for Lung Cancer: Current Understandings and Limitations. J. Clin. Med..

[143] Zhang W., Qin X., Zhang K., Ma J., Li M., Jin G., Liu X., Wang S., Wang B., Wu J. (2024). Microbial metabolite trimethylamine-N-oxide induces intestinal carcinogenesis through inhibiting farnesoid X receptor signaling. Cell. Oncol..

[144] Koeth R.A., Wang Z., Levison B.S., Buffa J.A., Org E., Sheehy B.T., Britt E.B., Fu X., Wu Y., Li L. (2013). Intestinal microbiota metabolism of L-carnitine, a nutrient in red meat, promotes atherosclerosis. Nat. Med..

[145] Zhou L., Zhang W., Fan S., Wang D., Tang D. (2024). The value of intratumoral microbiota in the diagnosis and prognosis of tumors. Cell Biochem. Funct..

[146] Zhang Y., Wu Q., Xu L., Wang H., Liu X., Li S., Hu T., Liu Y., Peng Q., Chen Z. (2021). Sensitive detection of colorectal cancer in peripheral blood by a novel methylation assay. Clin. Epigenetics.

[147] Yan X., Yang M., Liu J., Gao R., Hu J., Li J., Zhang L., Shi Y., Guo H., Cheng J. (2015). Discovery and validation of potential bacterial biomarkers for lung cancer. Am. J. Cancer Res..

[148] Liang Q., Chiu J., Chen Y., Huang Y., Higashimori A., Fang J., Brim H., Ashktorab H., Ng S.C., Ng S.S.M. (2017). Fecal Bacteria Act as Novel Biomarkers for Noninvasive Diagnosis of Colorectal Cancer. Clin. Cancer Res..

[149] Ma Y., Chen H., Li H., Zheng M., Zuo X., Wang W., Wang S., Lu Y., Wang J., Li Y. (2024). Intratumor microbiome-derived butyrate promotes lung cancer metastasis. Cell Rep. Med..

[150] Sivan A., Corrales L., Hubert N., Williams J.B., Aquino-Michaels K., Earley Z.M., Benyamin F.W., Lei Y.M., Jabri B., Alegre M.L. (2015). Commensal Bifidobacterium promotes antitumor immunity and facilitates anti-PD-L1 efficacy. Science (New York, N.Y.).

[151] Gopalakrishnan V., Helmink B.A., Spencer C.N., Reuben A., Wargo J.A. (2018). The Influence of the Gut Microbiome on Cancer, Immunity, and Cancer Immunotherapy. Cancer Cell.

[152] Bao J., Li L., Zhang Y., Wang M., Chen F., Ge S., Chen B., Yan F. (2022). Periodontitis may induce gut microbiota dysbiosis via salivary microbiota. Int. J. Oral Sci..

[153] de Andrade Calaça P.R., Bezerra R.P., Albuquerque W.W.C., Porto A.L.F., Cavalcanti M.T.H. (2017). Probiotics as a preventive strategy for surgical infection in colorectal cancer patients: a systematic review and meta-analysis of randomized trials. Transl. Gastroenterol. Hepatol..

[154] Zhu Z., Huang J., Zhang Y., Hou W., Chen F., Mo Y.Y., Zhang Z. (2024). Landscape of tumoral ecosystem for enhanced anti-PD-1 immunotherapy by gut Akkermansia muciniphila. Cell Rep..

[155] Yang J., Wei H., Zhou Y., Szeto C.H., Li C., Lin Y., Coker O.O., Lau H.C.H., Chan A.W.H., Sung J.J.Y., Yu J. (2022). High-Fat Diet Promotes Colorectal Tumorigenesis Through Modulating Gut Microbiota and Metabolites. Gastroenterology.

[156] Louis P., Flint H.J. (2017). Formation of propionate and butyrate by the human colonic microbiota. Environ. Microbiol..

[157] Hakozaki T., Richard C., Elkrief A., Hosomi Y., Benlaïfaoui M., Mimpen I., Terrisse S., Derosa L., Zitvogel L., Routy B., Okuma Y. (2020). The Gut Microbiome Associates with Immune Checkpoint Inhibition Outcomes in Patients with Advanced Non-Small Cell Lung Cancer. Cancer Immunol. Res..

[158] Yu H., Li X.X., Han X., Chen B.X., Zhang X.H., Gao S., Xu D.Q., Wang Y., Gao Z.K., Yu L. (2023). Fecal microbiota transplantation inhibits colorectal cancer progression: Reversing intestinal microbial dysbiosis to enhance anti-cancer immune responses. Front. Microbiol..

[159] Song Q., Gao Y., Liu K., Tang Y., Man Y., Wu H. (2024). Gut microbial and metabolomics profiles reveal the potential mechanism of fecal microbiota transplantation in modulating the progression of colitis-associated colorectal cancer in mice. J. Transl. Med..

[160] Chen W.D., Zhang X., Zhang Y.P., Yue C.B., Wang Y.L., Pan H.W., Zhang Y.L., Liu H., Zhang Y. (2022). Fusobacterium Nucleatum Is a Risk Factor for Metastatic Colorectal Cancer. Curr. Med. Sci..

[161] Chowdhury S., Castro S., Coker C., Hinchliffe T.E., Arpaia N., Danino T. (2019). Programmable bacteria induce durable tumor regression and systemic antitumor immunity. Nat. Med..

[162] Danino T., Prindle A., Kwong G.A., Skalak M., Li H., Allen K., Hasty J., Bhatia S.N. (2015). Programmable probiotics for detection of cancer in urine. Sci. Transl. Med..

[163] Brevi A., Zarrinpar A. (2023). Live Biotherapeutic Products as Cancer Treatments. Cancer Res..

[164] Wang H., Zheng L., Yang C., Jia L., Zhou R., Liu H., Dong Y., Xu X., Shi G., Yang J. (2025). Probiotic-mediated tumor microenvironment reprogramming with protease-sensitive interleukin-15 and photothermal therapy. Cell Rep. Med..

[165] Mei Y., Zhu J., Shao J., Li L., Liu F., Sha X., Yang Y., Shen J., Li R., Liu B. (2025). Engineered Mycobacterium smegmatis expressing anti-PD-L1/IL-15 immunocytokine induces and activates specific antitumor immunity. J. Immunother. Cancer.

[166] Si W., Liang H., Bugno J., Xu Q., Ding X., Yang K., Fu Y., Weichselbaum R.R., Zhao X., Wang L. (2022). Lactobacillus rhamnosus GG induces cGAS/STING- dependent type I interferon and improves response to immune checkpoint blockade. Gut.

[167] Rottinghaus A.G., Ferreiro A., Fishbein S.R.S., Dantas G., Moon T.S. (2022). Genetically stable CRISPR-based kill switches for engineered microbes. Nat. Commun..

